# Linking the duodenal microbiota to stunting in a cohort of undernourished Bangladeshi children with enteropathy

**DOI:** 10.1056/NEJMoa1916004

**Published:** 2020-07-23

**Authors:** Robert Y. Chen, Vanderlene L. Kung, Subhasish Das, Md. Shabab Hossain, Matthew C. Hibberd, Janaki Guruge, Mustafa Mahfuz, S. M. Khodeza Nahar Begum, M. Masudur Rahman, Shah Mohammad Fahim, Md. Amran Gazi, M. Rashidul Haque, Shafiqul Alam Sarker, R. N. Mazumder, Blanda Di Luccia, Kazi Ahsan, Elizabeth Kennedy, Jesus Santiago-Borges, Dmitry A. Rodionov, Semen A. Leyn, Andrei L. Osterman, Michael J. Barratt, Tahmeed Ahmed, Jeffrey I. Gordon

**Affiliations:** 1 Edison Family Center for Genome Sciences and Systems Biology, Washington University School of Medicine, St. Louis, MO 63110 USA; 2 Center for Gut Microbiome and Nutrition Research, Washington University School of Medicine, St. Louis, MO 63110 USA; 3 Department of Pathology and Immunology, Washington University School of Medicine, St. Louis, MO 63110 USA; 4 International Centre for Diarrhoeal Disease Research, Bangladesh (icddr,b), Dhaka 1212, Bangladesh; 5 Department of Pathology, Dr. Sirajul Islam Medical College, Dhaka 1217, Bangladesh; 6 Sheikh Russel National Gastroliver Institute and Hospital, Dhaka 1210, Bangladesh; 7 A. A. Kharkevich Institute for Information Transmission Problems, Russian Academy of Sciences, Moscow 127994, Russia; 8 Infectious and Inflammatory Disease Center, Sanford Burnham Prebys Medical Discovery Institute, La Jolla, CA 92037 US

**Keywords:** Childhood undernutrition, stunting, small intestinal microbiota, duodenal proteome, enteropathy

## Abstract

**BACKGROUND:**

Environmental enteric dysfunction (EED) is an enigmatic disorder of the small intestine postulated to play a role in childhood undernutrition, a pressing global health problem. Defining the incidence of EED, its pathophysiology, and its contribution to impaired linear and ponderal growth has been hampered by the difficulty in directly sampling the small intestinal mucosa and microbial community (microbiota).

**METHODS:**

Slum-dwelling Bangladeshi children aged 18±2 months, with linear growth-faltering (stunting) who failed a nutritional intervention underwent endoscopy to obtain duodenal biopsies and aspirates. Levels of 4077 plasma proteins and 2619 duodenal proteins were quantified in 80 children with histopathologic evidence of EED, and the abundances of bacterial strains in their duodenal microbiota were determined using culture-independent methods. Young germ-free mice, fed a Bangladeshi diet, were colonized with bacterial strains cultured from the duodenal aspirates.

**RESULTS:**

The absolute abundances of a shared group of 14 bacterial strains recovered from the duodenums of children with EED and not typically classified as enteropathogens were negatively correlated with linear growth (length-for-age Z-score;β=-0.38±0.12(SEM);ρ=-0.49;p=0.003), and positively correlated with duodenal proteins involved in immunoinflammatory responses. Representation of these 14 duodenal taxa was significantly different in fecal microbiota from EED versus healthy children (p<0.001;PERMANOVA). Gnotobiotic mice colonized with cultured EED-donor duodenal strains develop a small intestinal enteropathy.

**CONCLUSIONS:**

These results provide evidence of a causal relationship between components of the small intestinal microbiota, enteropathy and stunting and offer a rationale for developing therapeutics that target what must no longer remain terra incognita-the small intestinal microbiota. ClinicalTrials.gov identifier: NCT02812615

Environmental enteric dysfunction (EED) is a disease of the small intestine whose etiology is poorly understood. The condition was first described in the 1960s by pathologists examining biopsies taken from the proximal small bowel of adult Peace Corps volunteers who had lived in areas with high fecal-oral contamination and developed diarrhea and intestinal malabsorption (1). Histopathologic changes included diminution in the height and number of intestinal villi with associated loss of absorptive surface area, disruption of the epithelial barrier and a chronic inflammatory infiltrate.

Several reports have documented an association between altered small intestinal absorptive function, defined by dual-sugar permeability tests, asymptomatic infection with one or more enteropathogens and stunting (2,3). Stunting is associated with poor developmental outcomes including reduced intellectual capacity and impaired responses to oral vaccines (4-7). Despite considerable effort, neither nutritional interventions nor initiatives to improve water, sanitation and hygiene (WASH) practices have proven effective in reducing the incidence of stunting (8,9). These disappointing results have lent support to the notion that a subclinical form of ‘enteric dysfunction’ plays an important role in growth faltering (10).

Most studies of EED have relied on non-validated fecal or plasma biomarkers since esophagogastroduodenoscopy (EGD), a procedure with inherent risks, is rarely performed in undernourished pediatric populations. Consequently, there are conflicting data about the incidence of EED and its relationship to stunting (11). Gut microbial community development is impaired in children with undernutrition, and there is evidence supporting the hypothesis that perturbed microbiota development contributes to growth faltering (12-14). While most studies in undernourished children have focused on fecal microbial communities, recent data suggest a potential role for bacteria in the upper gastrointestinal tract (15).

The Bangladesh Environmental Enteric Dysfunction Study (BEED), conducted in an urban slum (Mirpur) in Dhaka (16) provides an opportunity to examine the role of the duodenal microbiota in the pathogenesis of EED and its relationship to stunting. This study has two components: (i) an interventional phase designed to test whether a nutritional intervention administered for 3 months would improve linear growth in 12-18-month-old children who were stunted or at risk for stunting (results reported in 17), and (ii), a ‘diagnostic’ component in those children whose growth faltering was refractory to the nutritional intervention and who, after informed consent was obtained, underwent EGD with duodenal biopsy. We have quantified levels of thousands of proteins in the plasma and duodenum of BEED children with a histopathologic diagnosis of EED and delineated the composition of their duodenal microbiota. The results reveal relationships between features of their duodenal microbiota, duodenal proteome, plasma proteome and linear growth. By culturing and sequencing the genomes of bacterial strains from duodenal aspirates, then introducing a consortium of these strains into young, germ-free mice fed diets representative of those consumed by children in Mirpur, we provide evidence that bacterial components of the small intestinal microbiota are causally related to EED.

## METHODS

### Study design, approval and sample collection

BEED was approved by the Ethical Review Committee at the icddr,b. Informed consent was obtained from each child’s mother/guardian. Children with length-for-age z-scores (LAZ) <-2 were classified as stunted, while those with LAZ >-2 and <-1 as at-risk for stunting. Both groups received one large (60-65g) egg, 150 mL of cow’s milk and multiple micronutrients 6 days/week for 3 months, plus an anti-helminthic (16). Children who failed to respond (defined as having a LAZ <-2 in the stunted group and LAZ >-2 but <-1 in the at-risk group), and without co-morbidities that cause undernutrition (16), qualified for EGD. Plasma, fecal samples, duodenal aspirates and duodenal biopsies were collected at EGD (**Table S1**) as were fecal samples from age-matched healthy children [LAZ, weight-for-length Z score (WLZ) >-1] living in Mirpur. None of the children undergoing EGD received antibiotics for at least 2 weeks prior to the procedure.

### SomaScan proteomic assay

This assay quantifies the abundances of 5284 proteins spanning a broad range of biological functions (18); 4077 plasma and 2619 duodenal proteins that passed quality control filtering were used for our analyses (see *Supplementary Methods*).

### Bacterial 16S rDNA-based analyses and enteropathogen detection

Duodenal aspirates and fecal samples were analyzed by sequencing PCR amplicons generated from variable region 4 of bacterial 16S ribosomal DNA (rDNA) genes. V4-16S rDNA reads were grouped into Amplicon Sequence Variants (ASVs); reads were normalized using DESeq2 (19), scaled by bacterial load and log10-transformed prior to analyses*.* A PCR-based assay was used to quantify duodenal and fecal abundances of 18 bacterial, viral, and protozoan enteropathogens (20).

### Gnotobiotic mouse experiments

Experiments were approved by the Washington University Animal Studies Committee. 5.5-week-old male germ-free C57BL/6J mice, fed a diet representative of that consumed by 18-month-old children living in Mirpur, were orally gavaged with a consortium of 39 bacterial strains cultured from the duodenums of children with histopathologic evidence of EED. Luminal contents were collected from different regions of the intestines of mice at the time of euthanasia, DNA was extracted and the absolute abundances of bacterial strains were defined (*Supplementary Methods*). Duodenal tissue was used for immunoassays and analysis of gene expression. Hematoxylin- and eosin-stained sections of intestinal segments were employed for histologic analysis.

### Statistical analysis

The strength of associations between duodenal protein modules, the abundances of plasma proteins, the absolute abundances of duodenal ASVs, histopathologic severity score, and LAZ were defined by Pearson correlation coefficient. Spearman rank correlation was used to identify relationships between LAZ and enteropathogen burden. Relationships between the duodenal proteome and absolute abundances of duodenal ASVs were assessed by Cross-Correlation Singular Value decomposition. Reported ‘p-values’ are unadjusted for multiple comparisons and adjusted q-values were calculated using false-discovery rate (FDR; 21). “Statistical significance” is described only when q-value <0.1. Point estimates and dispersion are reported as means and 95% confidence interval (CI), respectively, unless stated otherwise.

## RESULTS

### CLINICAL FEATURES OF CHILDREN WHO FAILED TO RESPOND TO NUTRITIONAL INTERVENTION

Children (n=525) were enrolled in the BEED study between July 2016 and July 2018. Informed consent was obtained for EGD of 110 children aged 18±2 months (mean±SD) who did not show significant improvements in linear growth (∆LAZ=0.03; 95% CI [-0.03, 0.08]; p=0.40) although they did manifest significant improvement in weight-for-age z-scores (∆WAZ=0.15; 95% CI [0.08, 0.22]; p<0.001) (**Table 1**).

**Table 1:** Clinical characteristics of children whose stunting was not improved by nutritional intervention.

	Failed nutritional intervention (n=110)	Biopsy-confirmed EED with available plasma and duodenal biospecimens (n=80)	n=110 vs n=80 comparison p-value§
**Demographic Features**			
Age (months)	18.4 ± 2.1	18.3 ± 2.1	0.82
Female - no. (%)	64 (58%)	48 (60%)	0.92†
WAMI index	0.58 ± 0.14	0.57 ± 0.13	0.64
Improved sanitation	79 (71.8%)	58 (72.5%)	0.92†
Improved source of drinking water	110 (100%)	80 (100%)	NA
Maternal education (years)	5 [1, 8]	5 [0, 7]	0.50‡
Income (USD/month)	153.1 [124.1, 212.0]	151.6 [124.1, 201.4]	0.90‡
Maternal height (cm)	149.0 ± 5.1	148.8 ± 5.5	0.81
Breastfeeding prior to intervention - no. (%)	100 (91%)	74 (93%)	0.90†
Breastfeeding after intervention - no. (%)	92 (84%)	70 (88%)	0.59†
**Response to Nutritional Intervention**			
∆LAZ	0.03 (-0.03, 0.08)	0.04 (-0.03, 0.11)	0.76
∆WAZ	0.15 (0.08, 0.22)*	0.13 (0.04, 0.23)*	0.76
∆WLZ	0.17 (0.07, 0.26)*	0.12 (0.00, 0.25)	0.57
**Anthropometry at Endoscopy**			
LAZ at endoscopy	-2.19 ± 0.82	-2.29 ± 0.86	0.49
WAZ at endoscopy	-1.76 ± 0.87	-1.85 ± 0.91	0.49
WLZ at endoscopy	-0.96 ± 0.91	-1.02 ± 0.93	0.64
**Duodenal Biopsy Histopathologic Score**			
No evidence of EED	6 (5.5%)	0 (0.0%)	NA
Mild	41 (37.3%)	34 (42.5%)	0.42
Moderate	13 (11.8%)	10 (12.5%)	0.53
Severe	50 (45.4%)	36 (45.0%)	0.13
**Fecal Biomarkers**			
𝛂1-antitrypsin (AAT, mg/g)	0.33 [0.17, 0.50]	0.33 [0.17, 0.50]	0.57
Myeloperoxidase (MPO, ng/mL)	2325.5 [799.6, 4569.1]	2006.0 [809.9, 4315.0]	0.75
Neopterin (NEO, nmol/L)	1116.5 [495.5, 2552.5]	1146 [506.3, 2575.3]	0.93
EE biomarker score	2.60 [1.52, 4.25]	2.77 [1.52, 4.24]	0.92

Values represent: mean ± standard deviation; number (percentage); mean difference (95% CI); median [interquartile range]. WAMI index = Water-sanitation-hygiene, Asset status, Maternal education status, and monthly Income (17). Statistically significant differences in characteristics between all children who did not improve with nutritional intervention (n=110) and those who had histologic evidence of EED as well as matched plasma and duodenal biospecimens available (n=80) were performed using an unpaired t-test unless otherwise noted.

* Statistically significant improvement in anthropometric measure as determined by paired t-test (p<0.05).

† Statistical significance determined using a Chi-squared test.

‡ Statistical significance determined using a Mann-Whitney U test.

§ See **Supplementary Table S2** for additional comparisons between sub-cohorts.

The duodenal biopsy used for histologic analysis was defined as having no (Grade 0, n=6), mild (Grade 1, n=41), moderate (Grade 2, n=13), or severe (Grade 3, n=50) EED based on a composite scoring system that considers the presence of infiltrating immune cells, villus atrophy and crypt hyperplasia (**Figure S1,** **Table 1**). There was no statistically significant correlation between LAZ and histopathologic severity defined in this way, or between LAZ and an ‘EE biomarker score’ that is based on fecal concentrations of three markers of intestinal inflammation and barrier disruption - myeloperoxidase, alpha-1-antitrypsin, and neopterin (p=0.79 and 0.36, respectively) (*Supplementary Methods*; **Table 2**).

Table 2Correlations between features of the plasma proteomes and duodenal microbiota of children with EED and their LAZ scores.

**Table:** 

Correlations between fecal biomarkers or histopathologic severity and LAZ (n=110 children)			
	Pearson ρ	Coefficient (95% CI)*†	p-value
Myeloperoxidase (MPO)	-0.19	-0.19 (-0.31, 0.00)	0.05
Alpha-1 antitrypsin (AAT)	-0.15	-0.13 (-0.29, 0.08)	0.11
Neopterin (NEO)	0.11	0.09 (-0.07, 0.24)	0.27
EE biomarker score	-0.09	-0.07 (-0.23, 0.08)	0.36
Histopathologic severity	-0.06	-0.06 (-0.27, 0.15)	0.57

**Table:** 

Correlations between features of the plasma proteome and LAZ (n=80 children)				
	Pearson ρ	Coefficient (95% CI)*†	p-value	FDR-adjusted q-value
Insulin-like growth factor 1 (IGF1)	0.53	0.62 (0.40, 0.84)	< 0.001	0.001
IGF acid-labile subunit (IGFALS)	0.51	0.59 (0.37, 0.81)	< 0.001	0.006
IGF binding protein 3 (IGFBP3)	0.48	0.56 (0.32, 0.80)	< 0.001	0.03
Procollagen C-endopeptidase Enhancer 2 (PCOLCE2)	0.40	0.46 (0.22, 0.70)	< 0.001	1
IGF binding protein 2 (IGFBP2)	-0.39	-0.46 (-0.70, -0.22)	< 0.001	1

**Table:** 

Correlations between features of the duodenal microbiota and LAZ (n=36 children)					
	Pearson ρ	Coefficient (95% CI)*†	p-value	FDR-adjusted q-value	Mean relative abundance (95% CI)*
*Streptococcus* sp.	-0.40	-0.31 (-0.54, -0.07)	0.02	0.10	45.2% (38.3%, 52.2%)
*Gemella* sp.	-0.38	-0.33 (-0.61, -0.06)	0.02	0.10	8.43% (5.82%, 11.04%)
*Neisseria subflava*	-0.41	-0.20 (-0.35, -0.05)	0.01	0.10	6.37% (4.04%, 8.71%)
*Haemophilus* sp.	-0.39	-0.20 (-0.37, -0.04)	0.02	0.10	8.02% (4.28%, 11.76%)
*Granulicatella elegans*	-0.47	-0.38 (-0.62, -0.13)	0.004	0.04	5.56% (4.29%, 6.82%)
*Veilonella* sp.	-0.44	-0.32 (-0.54, -0.10)	0.007	0.06	0.79% (0.57%, 1.00%)
*Rothia mucilaginosa*	-0.45	-0.31 (-0.53, -0.10)	0.006	0.06	2.37% (1.30%, 3.43%)
*Actinomyces* sp.	-0.37	-0.29 (-0.53, -0.04)	0.03	0.10	0.44% (0.33%, 0.54%)
*Leptotrichia* sp.	-0.49	-0.28 (-0.46, -0.11)	0.003	0.04	2.38% (1.45%, 3.32%)
*Prevotella melaninogenica*	-0.48	-0.28 (-0.45, -0.10)	0.003	0.04	1.55% (0.73%, 2.36%)
*Fusobacterium* sp.	-0.48	-0.27 (-0.44, -0.10)	0.003	0.04	0.92% (0.55%, 1.29%)
*Leptotrichia shahii*	-0.50	-0.35 (-0.56, -0.14)	0.002	0.03	0.46% (0.14%, 0.78%)
*Corynebacterium* sp.	-0.32	-0.22 (-0.45, 0.00)	0.06	0.10	0.18% (0.11%, 0.25%)
*Johnsonella* sp.	-0.36	-0.26 (-0.50, -0.03)	0.03	0.10	0.24% (0.14%, 0.33%)
Total bacterial load‡	-0.41	-0.32 (-0.56, -0.08)	0.01	0.10	33.0 (2.67, 63.3)
Total bacterial load of 'core group' taxa§	-0.49	-0.38 (-0.61, -0.15)	0.003	0.04	38.9 (34.1, 43.8)

* CI denotes 95% confidence interval

† Fecal biomarkers, plasma proteins, total bacterial load, and abundances of duodenal bacterial taxa were log-transformed and z-scored prior to determining their relationships with LAZ. Thus, coefficients represent the effect of a unit change in standard deviation of the independent variable on the dependent variable. Fecal biomarker concentrations are reported as their measured concentrations, prior to log-transformation or z-scoring.

‡ Total bacterial load represents the inverse of the fractional abundance of the *A. acidophilus* spike-in.

§ Total bacterial load of core group taxa represents the summed absolute abundances of the 14 bacterial ASVs found in > 80% of duodenal aspirates at a relative abundance of >0.01%.

Matched sets of plasma samples and duodenal biopsies were collected from 84 of the 110 children, 4 of whom had normal histology (**Table 1**, **Tables S1** and **S2**). There was sufficient fluid in the duodenal lumen of 38 of these 84 individuals to aspirate material for analysis of microbial community content; two had normal histology.

### THE PLASMA PROTEOME OF CHILDREN WITH EED

Because biopsies were not obtained prior to nutritional intervention, we could not confirm whether EED was present at enrollment. This consideration, together with the small number of children with a ‘normal’ biopsy and available plasma (n=4), and the fact that a single normal duodenal biopsy does not rule out the possibility of pathology at another site, led us to focus our analysis on post-intervention plasma samples from children with a histopathologic diagnosis of EED. The top five plasma proteins strongly correlated with LAZ were insulin-like growth factor 1 (IGF-1), IGF acid-labile subunit (IGFALS), IGF binding protein 3 (IGFBP-3), procollagen C endopeptidase enhancer 2 (PCOLCE2), and IGFBP-2 (**Table 2****; Table S3A**). Like IGFALS, IGFBP-3 stabilizes IGF-1, increasing its half-life and bioavailability, whereas IGFBP-2, which was negatively correlated with LAZ, sequesters IGF-1, inhibiting its growth-potentiating functions (22). PCOLCE2 influences bone formation by facilitating cleavage of the C-terminal propeptide of type I procollagen (23).

A linear model was created to examine the interrelationship between IGF-1 and LAZ, and the effect (interaction) of other members of the plasma proteome on this relationship (*Supplementary Methods*). Osteoprotegrin (OPG/TNFRSF11B), a decoy receptor for RANK ligand that promotes bone growth by inhibiting osteoclastic activity (24), and phosphate-regulating neutral endopeptidase (PHEX), the product of the gene responsible for X-linked hypophosphatemia and hereditary rickets (25), were among the top 5 plasma proteins with the most positive interactions with IGF-1 as related to LAZ (β=0.22±0.08, unadjusted p=0.005; β=0.25±0.08, unadjusted p=0.004, respectively) [**Table S4;** see *Supplementary Results*, **Figure S2**, **Table S5A-F** for comparisons of the plasma proteomes of the 80 children with EED prior to and after nutritional intervention, and of their age-matched healthy counterparts (n=21)].

### RELATIONSHIPS BETWEEN THE DUODENAL MICROBIOTA, PROTEOME AND GROWTH FALTERING

The composition of the microbial communities in duodenal aspirates recovered from the subset of 36 children with EED was quantified using culture-independent methods. For ethical reasons, duodenal specimens were not obtained from healthy children living in Mirpur. Shotgun sequencing of aspirate DNAs revealed that bacteria dominated community membership (**Online SupplementaryTable 2**). V4-16S rDNA sequence analysis revealed a total of 165 ASVs with relative abundances of at least 0.01% in one or more samples. A ‘core group’ of 14 bacterial taxa were present in ≥80% of the EED-associated aspirates (**Table S6**). PERMANOVA revealed no significant effects of breastfeeding status on duodenal bacterial composition (p>0.05; see *Supplementary Methods*).

We used an additional duodenal biopsy obtained from each of the 80 children that had not been used for histopathologic analysis to quantify expressed proteins. Our goal was to obtain a mechanistic definition of enteropathy that was more comprehensive than that provided by a hematoxylin- and eosin-stained section; we reasoned that such a definition could serve as a starting point for identifying features in the duodenal habitat that might correlate with duodenal microbiota composition and LAZ. Independent components analysis (ICA) is a method used to identify modules of co-expressed genes that belong to distinct biological pathways (26,27). Therefore, we used ICA to search for groups of duodenal proteins involved in inflammation, injury, and metabolic dysfunction that could underlie the enteropathy observed in BEED participants. Applying ICA to 2619 proteins in the 80 duodenal proteomic datasets (*Supplementary Methods*) allowed us to group 901 proteins into 14 modules containing between 63 and 183 proteins. Gene Ontology ‘Biological Process’ terms most enriched in each module are provided in **Online Supplementary DataTable 1**. Module 1 contained proteins enriched in GO terms related to neutrophil activation, acute inflammation and host immune responses against pathogens (**Table S7A,B**).

**Figure. 1:**
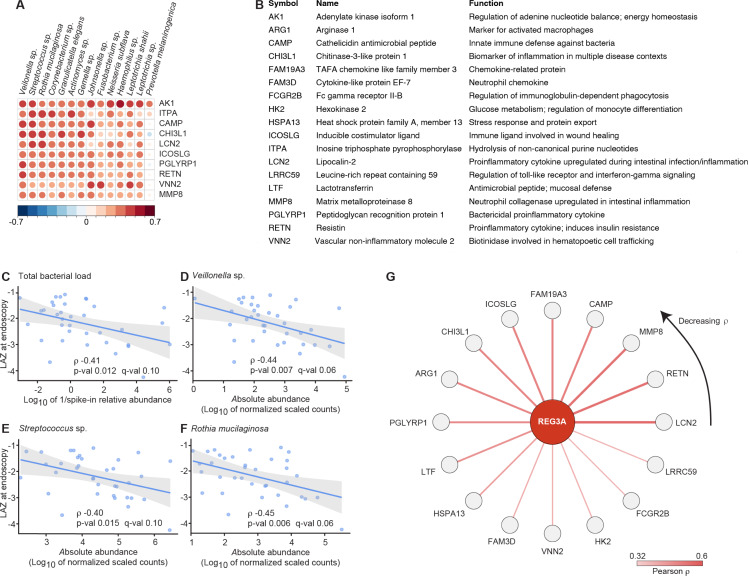
Correlations between components of the duodenal proteome, the absolute abundances of duodenal bacterial taxa and stunting. (**A**) The top 10 positive correlations between members of the 14 core taxa and duodenal proteins. The size and color of the circle represents the magnitude of the correlation (larger circle and darker color indicates stronger correlation). (**B**) Annotations of proteins shown in panels A and G. (**C-F**) Total duodenal bacterial load, and the abundances of the three organisms most positively correlated with duodenal inflammatory proteins are significantly negatively correlated with LAZ. The least-squares regression line is depicted in blue while shaded regions denote 95% confidence bands. (**G**) Star network of correlations between plasma REG3A and core taxa-associated duodenal proteins. As indicated by the color key, edge transparency/color corresponds to correlation strength (darker edges denote a stronger correlation). The duodenal protein with the strongest correlation, lipocalin-2 (LCN-2), is indicated by the tail of the arrowhead at 3 o’clock; duodenal proteins with progressively weaker correlations with REG3A are distributed in a counter-clockwise fashion from this position.

Cross-correlation singular value decomposition (CC-SVD) was performed to determine whether the absolute abundances of core group bacteria co-varied significantly with the abundances of duodenal proteins (28, *Supplementary Methods*). **Figure S3** lists the 50 proteins with the most significant positive correlations and the 50 having the most significant negative correlations with the absolute abundances of these core taxa. Twenty-two of the 100 proteins were members of ICA Module 1 (hypergeometric test of enrichment, p<0.001). **Figure 1A,B** summarizes the top 10 positive correlations, including two anti-microbial peptides [cathelicidin LL-37 and chitinase-3-like protein 1], plus an established fecal biomarker of intestinal inflammation, lipocalin-2 (LCN-2). In addition, the abundances of these taxa negatively correlated with a number of proteins produced by absorptive enterocytes (e.g., CDHR5, required for brush border formation; 29) (**Figure S3A,B**).

Total duodenal bacterial load and the abundances of 13 of the 14 core group taxa, including the three organisms most strongly correlated with duodenal inflammatory proteins in Module 1 (a member of the genus *Veillonella,* a *Streptococcus* sp. and *Rothia mucilaginosa*), were significantly negatively correlated with LAZ (FDR-corrected q<0.1, **Figure 1C-F**; **Table 2**; see **Table S3B** for duodenal proteins correlated with LAZ). Neither total bacterial load nor the abundances of one or more of these 13 taxa were significantly correlated with the severity of histopathology (linear regression p>0.05). qPCR assays of 18 enteropathogens commonly observed in the study population (20) revealed that their representation and abundances in duodenal aspirates (**Table S8A**) were not significantly correlated with LAZ (Spearman correlation, FDR corrected p>0.1).

We expanded our analysis to include the additional 44 children who provided plasma samples and duodenal biopsies but not aspirates (n=80). Analysis of plasma proteins that were significantly correlated with the 100 duodenal proteins associated with the 14 core taxa yielded (i) regenerating islet-derived protein 3-alpha (REG3A) which had the strongest correlation (p=6.9x10-6, FDR-corrected q=0.03), (ii) lipocalin-2 (q=0.09), and (iii) 6-pyruvoyltetrahydrobiopterin synthase (PTS, q=0.1), an enzyme involved in production of BH4, a cofactor for a number of enzymes including those involved in production of nitric oxide and several neurotransmitters. **Figure 1G** summarizes positive correlations between plasma REG3A and the core taxa-associated duodenal proteins; note that the strongest correlation is with duodenal lipocalin-2 (see **Figure S4** for duodenal proteins correlated with plasma LCN-2 and PTS).

Comparing the abundances of each of the 14 duodenal core taxa in fecal samples from healthy and EED children revealed that the relative abundance of a *Veillonella* sp., the bacterium most positively correlated with proteins involved in duodenal inflammation, was significantly elevated in EED fecal samples (p=0.001, FDR-corrected q=0.01). This organism has also been associated with stunting in a study of children living in Madagascar and the Central African Republic (15). An even greater difference could be appreciated when all 14 taxa were considered together (p<0.001; PERMANOVA) (**Figure S5**). In both EED and healthy children, the most frequently detected pathogens in the fecal microbiota were *E. coli* strains (EPEC, ETEC, EAEC), *Giardia* and *Campylobacter*. While there were no significant differences in the levels of any one enteropathogen between the two groups (p>0.05, Mann-Whitney), significantly greater pathogen diversity was detected in children with EED [4.6±2.0 versus 2.7±1.4 for healthy controls (mean±SD); p=0.002 (Mann-Whitney); **Table S8B**]. Neither the number of different pathogens detected in feces, nor the levels of any one of them significantly correlated with LAZ in children with EED (Spearman correlation FDR-corrected q>0.05).

### CULTURED DUODENAL BACTERIAL STRAINS FROM CHILDREN WITH EED TRANSMIT AN ENTEROPATHY

**Figure. 2:**
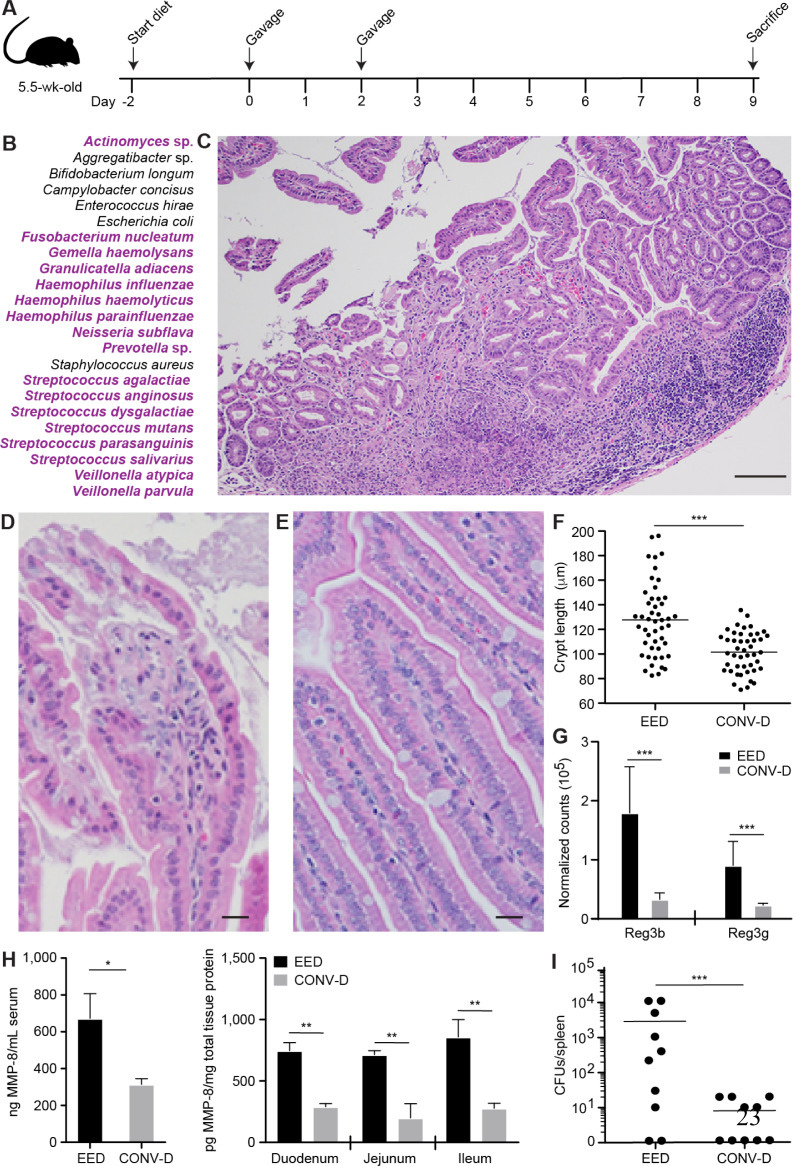
A defined consortium of cultured duodenal bacterial taxa from Bangladeshi children with EED transmits an enteropathy to gnotobiotic mice. (**A**) Experimental design. The majority of mice gavaged with the EED strains (12/16), and all CONV-D mice (n=10) were euthanized seven days after the final gavage (four mice gavaged with the EED consortium became moribund five days following the second gavage and were euthanized; at this time point they had lost 20% of their starting body weight compared to -4.7% ± 7.7% for all other animals receiving the consortium and -4.9 ± 5.3% for the CONV-D group). (**B**) Twenty-three bacterial strains that colonized animals at a relative abundance >0.01% at one or more locations along the intestine. See **Figure S6** for the biogeographical features of colonization along the length of the intestine. Strains belonging to the 14 core taxa detected in the duodenums of children with EED at the Genus level are highlighted in purple. (**C-E**) Hematoxylin- and eosin-stained sections showing representative histopathologic changes in the duodenal epithelium and lamina propria of mice colonized with the EED consortium (panels C and D) compared to CONV-D controls (panel E). See *Supplementary Results* and **Supplementary Table S12** for flow cytometry of immune cell populations. Scale bars, 100 µm in panel C, 25 µm in panels D and E. (**F**) Quantification of crypt length in the proximal 3 cm of the small intestine. The 10 best oriented crypts were measured (n=5 mice/treatment group). Each dot represents one measurement. Horizontal lines denote mean values. ***, p<0.001 (ANOVA). (**G**) Differential expression of duodenal Reg3β and Reg3γ as determined by DESeq2 (FDR-corrected q<0.001). (H) MMP-8 protein levels in serum and small intestine. *, p=0.01 (serum, unpaired t-test); **, p=0.005 (duodenum), p=0.002 (jejunum), p=0.001 (ileum) (2-way ANOVA with Sidak’s multiple comparisons test). (I) Bacterial translocation from the gut is increased in mice colonized with the EED consortium. Each dot represents a splenic homogenate from one mouse (two independent experiments; n=5 mice/experiment; mean values are shown as horizontal lines; ***, p<0.001). All translocated bacteria recovered from mice gavaged with the EED consortium were identified as *Escherichia coli* and *Enterococcus hirae* (the former lacks virulence-associated markers of diarrheagenic strains of *E. coli*). Rare *Enterococcus faecalis*, *Acinetobacter lwoffii*, and *Acinetobacter radioresistens* were recovered from the spleens of CONV-D controls.

Having demonstrated that a shared group of bacterial taxa present in duodenal microbiota of children with EED is associated with a proteomic signature of duodenal inflammation and growth-faltering, we developed a gnotobiotic mouse model to determine whether these taxa are causally related to the pathogenesis of their enteropathy. We first cultured 39 bacterial strains from duodenal aspirates, including 11 of the 14 ‘core group’ taxa (ASVs) (see **On-line Supplementary Table 3** and **Figure S6** for functional annotations of their genomes). The 39-member consortium was introduced into 5.5-week-old C57BL/6J ‘germ-free’ mice that had been reared under sterile conditions. Mice were fed an irradiated, animal protein-deficient diet representing the types and ratios of complementary foods typically consumed by 18-month-old children living in Mirpur (**Table S9**, see **Figure 2A** for experimental design). A control group of germ-free animals was colonized with cecal contents harvested from a conventionally-raised C57Bl/6J mouse [yielding ‘conventionalized’ (CONV-D) mice; n=10]. Three independent experiments were performed with the EED consortium (n=16 mice); the representation of members of the bacterial consortium was quantified by shotgun sequencing of DNA isolated from their duodenal, jejunal, ileal, cecal and colonic contents plus feces. The results revealed that 23 of the 39 strains, including 9 corresponding to core duodenal bacterial ASVs that negatively correlated with LAZ (including the *Veillonella* sp.), were detected at mean relative abundances >0.01% at one or more locations along the gastrointestinal tract (**Figure 2B**, **Figure S7, Table S10**).

Unlike CONV-D mice (or those maintained as germ-free), animals colonized with the EED consortium all displayed an inflammatory infiltrate in their small intestinal lamina propria dominated by mononuclear cells/lymphocytes, with associated disruption of the overlying epithelium and sloughing of epithelial cells from the upper portion of villi (**Figure 2C-E**). Crypt elongation, a regenerative response to epithelial damage, was the most consistent feature of architectural distortion (**Figure 2F**). In all animals, these histopathologic changes occurred in a patchy distribution along the length of the small intestine and spared the colon. Consistent with the innate immune response against microbes documented in children with EED, RNA-Seq analysis revealed that the bactericidal C-type lectins Reg3β and Reg3γ were among the most elevated transcripts in the duodenums of mice colonized with the EED consortium compared to CONV-D controls (**Figure 2G****, Table S11**; see *Supplementary Results* and **Table S12** for flow cytometry-based characterization of small intestinal immune responses). Matrix metalloproteinase-8 (MMP-8), a protein whose levels correlated with the absolute abundances of duodenal bacterial taxa in children with EED (**Figure 1G**), was significantly elevated in the sera and along the length of the small intestine of mice colonized with the EED consortium compared to CONV-D controls (**Figure 2H**). The observed small intestinal epithelial barrier disruption was also associated with decreased levels of mRNA transcripts for several intercellular tight junction components (**Table S11**) plus bacterial translocation into the systemic circulation, as evidenced by recovery of viable *E. coli* and *Enterococcus hirae* in the spleens of EED mice (**Figure 2I**). Together, these results provide evidence for a causal role for bacteria cultured from the duodenums of children with EED and the pathogenesis of their enteropathy.

## DISCUSSION

The BEED study provided an opportunity to assess the contribution of the proximal small intestinal microbiota to stunting in an undernourished Bangladeshi pediatric population whose linear growth faltering was unresponsive to a nutritional intervention. Endoscopy allowed us to establish a diagnosis of EED based on characteristic histopathologic changes in duodenal mucosal biopsies. Follow-up analyses of biospecimens uncovered a strong correlation between the absolute abundances of a group of 14 duodenal bacterial taxa and the degree of stunting in these children, and identified duodenal proteins whose abundances covary with the absolute abundances of these bacterial taxa. Plasma proteins whose levels correlate with features of the duodenal proteome together with the abundances of these 14 duodenal taxa in feces represent candidate biomarkers of the disease. A gnotobiotic mouse model provided evidence for a causal relationship between the duodenal microbiota and enteropathy.

These findings need to be extended in order to assess the degree to which they apply/generalize to other populations of children with stunting, including validation of candidate biomarkers in children with impaired versus healthy growth phenotypes. The small intestinal microbiota remains a ‘terra incognita’ and its relationship to the pathogenesis of gut barrier dysfunction, enteropathy and growth faltering is largely unexplored. Our results emphasize the need for less invasive small intestinal imaging and sampling techniques than those currently available in routine practice (30).

The lack of correlation between the histopathologic scoring system used here and LAZ is consistent with reports from other studies that employed more elaborate scoring schemes (31). Our findings underscore the value of characterizing the duodenal proteome and linking its features to components of the microbiota and in turn to linear growth. A next step will be to delineate the degree to the core EED duodenal taxa are represented in fecal biospecimens collected from other cohorts of healthy and stunted children, and thus clarify whether they could serve as accessible biomarkers of EED and the extent to which they function as pathobionts.

The gnotobiotic mouse model that provided evidence for a causal role between bacterial members of the proximal small intestinal microbiota and the pathologic features of EED opens up a number of opportunities; they include further dissection of interactions between members of the consortium of bacterial strains that lead to enteropathy, defining the factors that affect their fitness (a goal that may be facilitated by the *in silico* metabolic reconstructions of their nutrient requirements presented in **Figure S6** and **On-line Supplementary Table 3**), exploration of the mechanisms by which they affect mediators of linear growth, and development of prebiotic, probiotic, synbiotic or pharmacologic therapeutic candidates.

### SUPPORT

This work was supported by the Bill & Melinda Gates Foundation. R.Y.C. is a member of the Medical Scientist Training Program which is supported by NIH grant GM007200. Histochemical and immunohistochemical processing of tissue sections was performed at the Washington University Digestive Diseases Research Core Center funded by NIH P30 DK052574. J.I.G. is the recipient of a Thought Leader Award from Agilent.

## Author affiliations

The Edison Family Center for Genome Sciences and Systems Biology (RYC, VLK, MCH, JG, BDL, KA, EK, JS-B, MJB, JIG), Center for Gut Microbiome and Nutrition Research (RYC, VLK, MCH, JG, KA, MJB, JIG) and Department of Pathology and Immunology (VLK, MJB, JIG), Washington University School of Medicine, St. Louis, MO 63110 USA; International Centre for Diarrhoeal Disease Research, Bangladesh (icddr,b), Dhaka 1212, Bangladesh (SD, MdSH, MM, SMKNB, MMR, SMF, MdAG, MRH, MSAS, RNM, KNB, TA); Department of Pathology, Dr. Sirajul Islam Medical College, Dhaka 1217, Bangladesh (NB); Sheikh Russel National Gastroliver Institute and Hospital, Dhaka 1210, Bangladesh (MMR); A. A. Kharkevich Institute for Information Transmission Problems, Russian Academy of Sciences, Moscow 127994, Russia (DAR, SAL); and Infectious and Inflammatory Disease Center, Sanford Burnham Prebys Medical Discovery Institute, La Jolla, CA 92037 USA (DAR, SAL, ALO).
