# Supplementary material for: Linking the duodenal microbiota to stunting in a cohort of undernourished Bangladeshi children with enteropathy
Source: N Engl J Med. Author manuscript; Available in PMC 2020 Jul 23. (PMC7289524; doi:10.1056/NEJMoa1916004)
Supplement: Supplementary file 1 [file NEJM-2020-1916004-s1.pdf]

ONLINE SUPPLEMENT

TABLE OF CONTENTS

Page

List of Investigators 5

SUPPLEMENTARY METHODS 6

HUMAN STUDIES 6

Sample collection 6

Assessing histopathologic severity of EED 6

Fecal biomarkers associated with EED 6

Breastfeeding 7

Processing of plasma samples for proteomic analyses 7

Processing of duodenal biopsy samples for proteomic analyses 8

Bacterial 16S rDNA analyses 8

Shotgun sequencing of duodenal aspirates and fecal samples 9

Quantifying pathogen burden by multiplex PCR 10

Recovering, sequencing and annotating bacterial strains from duodenal aspirates 10

GNOTOBIOTIC MOUSE STUDIES 11

Design and preparation of a representative Mirpur diet 11

Animal Husbandry 12

Community profiling by sequencing (COPRO-Seq) 13

Assays of MMP-8 13

Duodenal RNA-Seq 13

Flow cytometry 14

Assaying spleens for viable bacteria 15

STATISTICAL ANALYSIS 15

Effects of nutritional intervention on ponderal and linear growth 15

Relationship between histopathologic score and linear growth 16

Relationship between fecal biomarkers and linear growth 16

Relationship between a fecal 'EE biomarker score' and LAZ 16

Comparative analyses of the plasma proteomes of healthy children or children with EED 17

Relating fecal biomarkers and plasma proteins to LAZ 17

Identifying modules of co-expressed duodenal proteins 18

Cross-correlation singular value decomposition (CC-SVD) analysis 19

Analysis of the representation of duodenal 'core' taxa in fecal samples from healthy children or children with EED 20

DATA DEPOSITION 20

SUPPLEMENTARY RESULTS 20

Comparison of the plasma proteomes of children with EED prior to and after nutritional intervention and of their healthy counterparts 20

Characterization of immune cell populations in gnotobiotic mice colonized with the EED donor-derived bacterial consortium 21

SUPPLEMENTARY FIGURES 23

Figure S1 – Scoring system for grading severity of histopathologic changes in duodenal biopsies 23

Figure S2 – Plasma proteins whose abundances are significantly different between children living in Mirpur with EED, prior to and after their nutritional intervention, and children from Mirpur judged to have healthy growth phenotypes. 24

Figure S3 – CC-SVD analysis of duodenal proteins and duodenal bacterial taxa 26

Figure S4 - Correlations between plasma LCN-2 or PTS and duodenal proteins 32

Figure S5 – Relative abundances of core duodenal taxa in the fecal microbiota of children living in Mirpur who have healthy growth phenotypes (n=27) and those with EED (n=48). 33

Figure S6 – Binary phenotype matrix of *in silico* predictions of metabolic functions of 39 cultured bacterial strains recovered from BEED duodenal aspirates 34

Figure S7 – Analysis of the distribution of bacterial strains along the length of the intestines of gnotobiotic mice 36

SUPPLEMENTARY TABLES 37

Table S1 – Biospecimens analyzed from BEED participants and from healthy controls 37

Table S2 – Clinical characteristics of subgroups in the BEED study 37

Table S3 – Correlations between plasma or duodenal proteins and LAZ. 37

Table S4 – Results of linear modeling of interactions between IGF-1 and plasma proteins/fecal biomarkers on LAZ 37

Table S5 - Analysis of the plasma proteomes of children living in Mirpur who had healthy growth or BEED children who were stunted or at risk for stunting and failed nutritional intervention... 38

Table S6 – Relative abundances of bacteria taxa (ASVs) and total bacterial load in duodenal aspirates from BEED children. 38

Table S7 – Duodenal proteins and assigned GO Biological Process terms in Module 1 38

Table S8 – qPCR assays for enteropathogens 39

Table S9 – Mirpur-18 diet given to gnotobiotic mice 39

Table S10– Distribution of members of the bacterial consortium along the length of the gastrointestinal tracts of recipient gnotobiotic mice 39

Table S11 – Differential expression of genes in the duodenums of gnotobiotic mice colonized with the cultured bacterial consortium from children with EED versus CONV-D controls 39

Table S12 – Flow cytometric analysis of immune cell populations present in the small intestinal epithelium and lamina propria plus mesenteric lymph nodes of gnotobiotic mice colonized with the EED bacterial consortium and CONV-D controls. 39

ONLINE SUPPLEMENTARY MATERIALS 39

Online Supplementary Data Table 1 - Duodenal proteome module membership in children with EED and GO Biological Process enrichment for each module. 39

Online Supplementary Data Table 2 – Duodenal aspirate microbial community composition of children with EED determined using MEtaPhlAn2. 40

Online Supplementary Data Table 3 – Annotation of the genomes of 39 bacterial strains cultured from duodenal aspirates obtained from Mirpur children with EED. 40

SUPPLEMENTARY REFERENCES 40

List of Investigators

Robert Y. Chen, B.S.[1,2]*, Vanderlene L. Kung, M.D., Ph.D.[1,2,3]*, Subhasish Das, M.B.B.S., M.P.H[4], Md. Shabab Hossain, M.B.B.S.[4] , Matthew C. Hibberd, Ph.D.[1,2,3], Janaki Guruge, Ph.D.[1,2], Mustafa Mahfuz, M.B.B.S., MPH[4], S. M. Khodeza Nahar Begum, M.B.B.S., M.D.[5], M. Masudur Rahman, M.B.B.S., M.D.[6], Shah Mohammad Fahim, M.B.B.S., M.P.H.[4], Md. Amran Gazi, M.Sc.[4], M. Rashidul Haque, M.B.B.S., Ph.D.[4], Shafiqul Alam Sarker, M.D., Ph.D.[4], R. N. Mazumder, M.B.B.S., M.D.[4], Blanda Di Luccia, Ph.D.[1,3], Kazi Ahsan, M.B.B.S., M.P.H.[1,2], Elizabeth Kennedy, B.S.[1], Jesus Santiago-Borges, B.S.[1], Dmitry A. Rodionov, Ph.D.[7,8], Semen A. Leyn, Ph.D.[7,8], Andrei L. Osterman, Ph.D.[8], Michael J. Barratt, Ph.D.[1,2,3], Tahmeed Ahmed, M.B.B.S., Ph.D.[4#], Jeffrey I. Gordon, M.D.[1,2,3 #+]

[*]Contributed equally; [#]co-senior authors

[1]Edison Family Center for Genome Sciences and Systems Biology, Washington University School of Medicine, St. Louis, MO 63110 USA

[2]Center for Gut Microbiome and Nutrition Research, Washington University School of Medicine, St. Louis, MO 63110 USA

[3]Department of Pathology and Immunology, Washington University School of Medicine, St. Louis, MO 63110 USA

[4]International Centre for Diarrhoeal Disease Research, Bangladesh (icddr,b), Dhaka 1212, Bangladesh

[5]Department of Pathology, Dr. Sirajul Islam Medical College, Dhaka 1217, Bangladesh

[6]Sheikh Russel National Gastroliver Institute and Hospital, Dhaka 1210, Bangladesh

[7]A. A. Kharkevich Institute for Information Transmission Problems, Russian Academy of Sciences, Moscow 127994, Russia

[8]Infectious and Inflammatory Disease Center, Sanford Burnham Prebys Medical Discovery Institute, La Jolla, CA 92037 US

SUPPLEMENTARY METHODS

HUMAN STUDIES

Sample collection

Sample collection procedures have been described previously (1). Five mL of venous blood was collected (S-Monovette 7.5 mL tube, Sarstedt) just prior to endoscopy, transported to the laboratory at 4 $^{\circ}$C, centrifuged to collect plasma; plasma was stored in aliquots at -80 $^{\circ}$C. [Celiac disease was excluded by measuring circulating levels of tissue transglutaminase IgA]. Duodenal aspirate samples were collected from the second portion of the duodenum using sterile Endoscopic Retrograde Cholangiopancreatography (ERCP) catheters. Several distal duodenal biopsies were taken (1). A duodenal biopsy was flash frozen in liquid nitrogen and preserved at -80 $^{\circ}$C for subsequent proteomic analysis. Fecal samples were collected immediately prior to endoscopy, aliquoted into 2 mL cryovials, placed in liquid nitrogen within 20 minutes of production and subsequently stored at -80 $^{\circ}$C until further processing.

Assessing histopathologic severity of EED

An operational categorization of EED was developed for this study based on three histopathologic features evident in hematoxylin- and eosin-stained tissue sections: infiltration of inflammatory cells in the lamina propria, blunting or atrophy of small intestinal villi, and hyperplasia or elongation of crypts of Lieberkuhn. Because inflammatory infiltration takes place prior to structural changes of either villus atrophy or crypt hyperplasia, mild EED (Grade 1) refers to the sole presence of inflammatory infiltrates in the lamina propria. Moderate EED (Grade 2) refers to any one of the two structural changes, i.e. either villi or crypts, in addition to inflammatory infiltration in the same biopsy sample. Severe EED (Grade 3) refers to the presence of structural changes involving both villi and crypts in addition to inflammatory infiltration in the same duodenal biopsy sample. Biopsies without any of these three features were considered as having no evidence of EED (Grade 0).

Fecal biomarkers associated with EED

Fecal concentrations of myeloperoxidase (MPO; Alpco), alpha-1-antitrypsin (AAT; Biovendor), and neopterin (NEO; GenWay Biotech) were measured using commercially available enzyme-linked immunosorbent assays (ELISAs) as previously described (1).

Breastfeeding

Mothers of BEED study participants were interviewed prior to and after nutritional intervention to ascertain their 'breastfeeding status', e.g. a binary metric representing whether or not the child was breastfeeding at the time of the interview. PCA parameterized by the 14 core taxa followed by PERMANOVA was performed on the 36 children with histopathologic evidence of EED to determine whether breastfeeding status was a significant determinant of duodenal community bacterial composition.

Processing of plasma samples for proteomic analyses

The SomaScan 5K Proteomic Assay plasma/serum kit (SomaLogic) was used to measure 5,284 proteins in plasma samples (50 µL aliquots) according to the manufacturer's protocol. Proteins were tagged with NHS-biotin reagent, captured as a SOMAmer (protein-specific aptamer) /protein complex immobilized on streptavidin beads, cleaved, denatured, eluted and hybridized to a custom Agilent DNA microarray. Microarrays were scanned with an Agilent SureScan instrument at 5 µm resolution and the Cy3 fluorescence readout was quantified. Raw fluorescence signal values from each SOMAmer reagent were processed using SomaLogic's SomaScan standardization procedures, including steps for hybridization normalization, plate scaling, median scaling and final SOMAmer calibration, each of which generates a SomaScan '.adat' format data file. The final .adat file was log2-transformed, quantile-normalized and filtered to remove all non-human SOMAmer reagents. An additional filter was applied to remove SOMAmer reagents with a probe-wise variance <0.07 to ensure protein abundances were within the limit of detection, resulting in quantitative abundance data for 4,077 plasma proteins used for all downstream analyses. We used singular value decomposition (SVD) to remove batch-associated variance from the SOMAmer fluorescence values. The standardized, transformed, normalized, filtered, and batch-corrected signal values were employed for comparisons between the plasma proteomes of anthropometrically healthy children and children with EED, as well as for pre- versus post-nutritional intervention

comparisons. For all other analyses utilizing measured abundances of plasma proteins, batch-correction was not performed. Note that the SomaScan platform contains aptamers with redundancies against several proteins. The reasons for these redundancies include capturing different isoforms, detecting proteolytic cleavage products or providing an internal control for probe specificity.

Processing of duodenal biopsy samples for proteomic analyses

Protein extraction from each duodenal biopsy was accomplished by adding 100 μL of ice-cold T-Per tissue protein extraction buffer (Thermo Scientific) plus Complete Ultra protease inhibitor (Roche). Samples were homogenized on ice using a disposable manual pestle until no tissue fragments were visible, and centrifuged at 14,000 x g for 10 min at 4 ^{o}C. Total protein concentration in the resulting supernatants was quantified using Micro BCA Protein Assay Kit (Thermo Scientific). Protein concentrations in all samples were normalized to 200 μg/mL with PBS.

The SomaScan 5K Proteomic Assay CEL/tissue kit was used to measure 5,284 proteins in duodenal lysate samples (50 μL aliquots of a 200 μg/mL mixture) according to the manufacturer's protocol. Fluorescence values for each SOMAmer reagent were standardized, transformed, normalized, and filtered in the same fashion as the plasma proteins. Principal components analysis (PCA) revealed batch effects that were corrected for using the 'removeBatchEffects' function from the R package *limma* (2). The resulting standardized, transformed, normalized, filtered, and batch-corrected signal values for 2,619 duodenal proteins were used for all downstream analyses.

Bacterial 16S rDNA analyses

Prior to isolating DNA from duodenal aspirates, 1.1 x 10^6 *Alicyclobacillus acidiphilus* cells were added to a 100 μL aliquot of an aspirate sample as a spike-in quantitative control. The material was then centrifuged at 5000 x g for 10 min at 10 ^{o}C in a 2 mL v-bottom tube (Axygen). All but 50 μL of the resulting supernatant was removed (to avoid disturbing the pellet). The pellet was then resuspended in the residual supernatant by pipetting, followed by digestion with proteinase K (Thermo Fisher Scientific; final concentration, 1 μg/μL reaction; incubation for 10 hours at 65 ^{o}C, followed by 10-minutes at 95 ^{o}C

to inactivate the enzyme). DNA from the resulting duodenal aspirate material, and from fecal samples (3), were subsequently isolated and V4-16S rDNA amplicon libraries were generated as described previously (3).

Amplicon libraries were sequenced (2x250 nt paired-end reads; Illumina MiSeq instrument) to a depth of $3.9 \pm 2.3 \times 10^4$ reads/duodenal aspirate and 4.4 ± 7.6 10^4 (mean±SD) reads/fecal sample, yielding a mixture of phased amplicons in two different orientations to achieve optimal base balance. The 'bbduk.sh' and 'repair.sh' tools in bbtools (37.02; https://sourceforge.net/projects/bbmap/) were used to orient the amplicons, trim primer sequences, and ensure proper read pairing. Pre-processed data were analyzed using the R (3.5.1) implementation of DADA2 (1.8.0; (4)) to identify and quantify ASVs. Taxonomic assignments of ASVs were performed using the DADA2 'assignTaxonomy' tool and the GreenGenes training set (version 13.8).

For duodenal aspirates, ASVs were agglomerated by phylogenetic distance using the *phyloseq* 'tip_glom' tool (tree height of < 0.2), and bacterial loads in duodenal aspirates were calculated by dividing total 16S rDNA amplicon sequencing reads not mapped to *A. acidiphilus* by *A. acidiphilus* reads (5,6). Counts were normalized using DESeq2 (7), scaled by bacterial load, to obtain the absolute abundance of each agglomerated ASV, and log10-transformed to obtain the final ASV absolute abundance data used for downstream analyses.

For the analysis of fecal microbiota of healthy children or children with EED, ASVs were agglomerated using 'tip_glom' and normalized using DESeq2 in the same fashion as ASVs from the duodenal aspirates. Agglomerated and normalized fecal ASV abundances were then transformed using 'varianceStabilizingTransformation' from the *DESeq* package (7), producing the compositional abundance data used for downstream analyses.

Shotgun sequencing of duodenal aspirates and fecal samples

DNA was extracted from duodenal aspirates and fecal aspirates, quantified (Qubit), and normalized to a concentration of 0.75 ng/mL. Libraries were generated from each sample using the Nextera XT kit (Illumina). Barcoded libraries were pooled and sequenced on an Illumina NextSeq instrument (2x150 nt

paired end reads; 12.5 ± 3.2 x 10^6 reads/duodenal aspirate; 10.2 ± 1.1 x 10^6 (mean ± SD) reads/fecal sample). After quality filtering with *Sickle* (v1.33) and trimming of Nextera adapter sequences with *cutadapt* (v1.16), taxonomic profiles were generated using MetaPhlAn2 (v2.0) (8). *Bowtie2* (v2.3.4.1) and the hG19 build of the *H. sapiens* genome were employed to identify and remove host sequences prior to further processing.

Quantifying pathogen burden by multiplex quantitiative PCR

Nucleic acids were isolated from duodenal aspirates and fecal samples (3) and the concentration normalized to 2 ng/µL. Levels of bacterial, viral, and protozoal gastrointestinal pathogens were determined using a microfluidic-based digital PCR system with 96.96 Dynamic Arrays (Fluidigm). TaqMan primers and probes were used to construct assays for 18 different enteropathogens. qPCR and data analysis methods are described in ref. (9). Reported values are absolute measurements of DNA mass in pg (all bacterial and protists) and copy number (all RNA viruses). Adenovirus-infected cells were used as the standard for the Adenovirus target; values reported for Adenovirus are a ratio of mass of viral DNA per cell lysate mass. Values were log-transformed prior to correlation analysis.

Recovering, sequencing and annotating bacterial strains from duodenal aspirates

All steps for culturing bacterial strains were performed in anaerobic chambers (Coy Laboratory Products). Duodenal aspirate samples (volumes ranged from 300 to 500 µL) were thawed. A 100 µL aliquot was taken for nucleic acid extraction, while the remaining volume was divided evenly into fourths to streak on BHI agar supplemented with 10% defibrinated horse blood, with and without vancomycin (10 µg/mL). Plates were incubated at 37 ºC for 2-3 days under anaerobic (5% H_2, 20% CO_2, 75% N_2) and microaerophilic (5% O_2, 5% CO_2, 90% N_2) conditions. Colonies were re-struck and initially identified by Matrix-Assisted Laser Desorption Ionization Time-of-Flight (MALDI-TOF) mass spectrometry (Vitek MS; bioMérieux Clinical Diagnostics). Cells were sub-cultured in LYBHI broth (BHI broth supplemented with 0.05% L-cysteine HCl and 0.5% yeast extract) for anaerobic colonies and BHI broth for microaerophilic colonies. Broth cultures were incubated for 1-2 days. Multiple stock vials were prepared

in PBS/15% glycerol and stored at -80 ºC. DNA was extracted from individual strains, quantified (Qubit), and normalized to a concentration of 0.75 ng/mL. Libraries were generated from each sample using the Nextera XT kit (Illumina). Genomes were assembled using *SPAdes* (10) under default parameters, and annotated with *Prokka* (11) and *RAST* (12). Species assignments were made using *CheckM* (13). Unique species were identified by calculating pairwise ANI (average nucleotide identity) values between genomes, using an ANI threshold of greater than 95% for species demarcation. Based on these results, we selected 39 cultured sequenced strains representative of the bacterial taxa identified by V4-16S rDNA amplicon sequencing and MALDI-TOF. These organisms were clonally-arrayed into multi-well plates. Monocultures of the organisms were combined in equivalent amounts, as assessed by OD_{600} values, to create a defined consortium for gavage into gnotobiotic mice.

In silico reconstructions of selected mcSEED metabolic pathways were based on functional gene annotation and prediction using homology-based methods and genome context analysis (14,15). Reconstructions were represented as a binary phenotype matrix (BPM) where for amino acids and B vitamins, “1” denotes a predicted prototroph and “0” an auxotroph, for carbohydrates, “1” and “0” refer to a strain’s predicted ability or inability, respectively, to utilize the indicated mono-, di- or oligosaccharide, and for fermentation end products (short chain fatty acids), a “1” and “0” indicate a strain’s predicted ability/inability to produce the indicated compound, respectively.

GNOTOBIOTIC MOUSE STUDIES

All mouse experiments were performed using protocols approved by Washington University Animal Studies Committee.

Design and preparation of a representative Mirpur diet

The composition of this diet was based on Bangladeshi complementary feeding practices for 18-month-old Mirpur children, including quantitative 24-hour dietary recall surveys conducted at the Mirpur site as part of the MAL-ED study (16). A pelleted version of this diet was manufactured for gnotobiotic mouse studies by Dyets Inc. Rice (parboiled, long grain) and red lentils (masoor dal) were each cooked

separately with an equal weight of water at 100 ºC in a steam-jacketed kettle until 'par-cooked' (grains cooked, but still firm) and then set aside. Fresh market white potatoes, spinach and yellow onions were washed, chopped in a vertical cutter mixer and cooked in the kettle without added water at 70 ºC until soft. Sweet pumpkin (Calabaza variety) was cut and boiled in the steam-jacketed kettle until soft, and then strained. At this point, all of the cooked ingredients were combined, soybean oil, salt, turmeric and garlic were added (see **Table S8** for quantities of ingredients). The resulting diet was mixed extensively, spread on trays, dried overnight at 30 °C, and pelleted by extrusion (½" diameter; California Pellet Mill, CL5). Dried pellets were aliquoted into ~250g portions, placed in a paper bag with an inner wax lining, which was placed in a plastic bag. Bags were subsequently vacuum sealed and their contents sterilized by gamma irradiation (30-50 kGy; Sterigenics). Sterility was verified by culturing irradiated pellets in Brain Heart Infusion (BHI) broth, Nutrient broth, and Sabouraud-dextran broth (all from Difco) for one week at 37 °C under aerobic conditions, and in Tryptic Soy broth (Difco) under anaerobic conditions (atmosphere of 5% H_2, 20% CO_2, 75% N_2). Additionally, cultures of all diets were plated on BHI agar supplemented with 10% horse blood (Difco). The irradiated diet pellets had an energy density of 4.88 kcal/g and were composed of 10.5% protein, 21.1% fat, and 0.42% fiber (Nestlé Purina Analytical Laboratories).
All diets were stored at -20 °C prior to use.

Animal Husbandry

Mice were housed in plastic flexible film gnotobiotic isolators (Class Biologically Clean Ltd.) at 23ºC under a strict 12-hour light cycle (lights on a 0600h). Male germ-free C57BL/6J mice were initially weaned onto an autoclaved, low-fat, high-plant polysaccharide chow that was administered *ad libitum* (Diet 2018S, Envigo). Animals were maintained on this diet until 3 days prior to the beginning of experiments. At 5.5 weeks of age, the defined consortium of 39 sequenced bacterial strains obtained from children with EED, or intact uncultured microbiota recovered from the cecum of an adult conventionally-raised C57BL/6 mouse was administered using a disposable sterile gavage needle (n=5 mice/cage/treatment group/experiment). All animals were weighed every 2 days and euthanized by cervical dislocation without prior fasting at the end of the study.

Community profiling by sequencing (COPRO-Seq)

The distribution of bacterial strains along the length of the gastrointestinal tracts of mice gavaged with the 39 cultured strains was defined by COPRO-Seq (17). Briefly, DNA was isolated from luminal contents harvested at the time of euthanasia from the duodenum (defined as proximal third of the small intestine), jejunum (middle third), ileum (distal third), cecum, colon, and feces. COPRO-Seq libraries were prepared; the barcoded libraries were quantified (Qubit dsDNA HS kit), pooled and then subjected to multiplex sequencing [Illumina NextSeq instrument; unidirectional 75 nt reads; $2.1 \pm 0.2 \times 10^6$ reads/sample (mean ± SD)]. Reads were demultiplexed and mapped to the reference genomes of community members plus 2 "distractor" genomes (*Bacteroides fragilis* NCTC 9343 and *Clostridium perfringens* ATCC13124). The proportion of reads mapping to "distractor" genomes in each sample was used to set a conservative threshold cutoff (mean + 2 SD), indicating the presence/absence of an organism in the community on a per-sample basis. Normalized counts for each bacterial strain in each sample were used to produce a relative abundance table.

Assays of MMP-8

Total protein was extracted from duodenum, jejunum, and ileum using the same procedure described above for human duodenal biopsies. Levels of MMP-8 in these intestinal segments and in the serum of mice colonized with the EED consortium, and in CONV-D controls, were assayed using MILLIPLEX MAP Mouse MMP Magnetic Bead Panels (MilliporeSigma) following the manufacturer's instructions.

Duodenal RNA-Seq

Total RNA was isolated from 10 mg of flash frozen tissue taken 6 cm from the gastroduodenal junction of mice euthanized 9 days after the initial gavage with the EED consortium or the cecal contents from a conventionally-raised C57Bl/6J mouse (RNeasy Plus Universal Mini Kit; Qiagen). Total RNA quality was checked with an Agilent Bioanalyzer 2100 using RNA 6000 Pico Chips (Agilent). For each sample, cDNA was synthesized from 10 ng total RNA using the SMARTer Ultra Low Input RNA for Illumina Sequencing – HV kit (Clontech). cDNA was sheared to 200-500 bp with a Covaris AFA system. A library was constructed following the Clontech "*adapted Nextera (Illumina) DNA sample preparation protocol*

for use only with SMARTer ultra-low DNA kit for Illumina sequencing." Samples were sequenced on an Illumina NextSeq 500 instrument using the High-Output 150 v2 Kit to generate 75-nucleotide paired-end reads [21.2 ± 9.3 x 10^6 (mean ± SD) reads/sample]. Reads were aligned to the Ensembl release 89 mouse primary assembly with *STAR* v2.5.3a (18). Gene counts were derived from the number of uniquely aligned unambiguous reads using featureCounts (Subread version 1.4.6-p5) (19). Sequencing performance in terms of total number of aligned reads, total number of uniquely aligned reads and gene body coverage was determined using RSeQC version 2.6.2 (20).

Gene counts were imported into the R/Bioconductor package DESeq2 (7) and normalized using default settings. Genes whose normalized counts added up to less than one across all samples were removed. Differential expression analysis was performed using DESeq2 (7). Differentially expressed genes were defined as those with an FDR-adjusted $p<0.10$ and fold-difference >1.25.

Flow cytometry

Small intestines were flushed to remove luminal contents. Intestines were opened lengthwise and gently agitated at 23 ºC for 20 minutes in Hanks Balanced Salt Solution (HBSS) supplemented with 10% bovine calf serum, 5 mM EDTA and 15 mM HEPES. Intestines were vortexed and supernatants were separated from tissue fragments by careful decantation. A second round of gentle agitation and vortexing of the pellets in the same buffer was performed and the supernatants from each round were combined and used for staining of intraepithelial lymphocytes. The tissue was then rinsed with HBSS prior to digestion with Collagenase IV (Sigma) in complete RPMI-10 for 40 minutes at 37 °C with gentle agitation. Digests were filtered through 100 μm mesh (BD Biosciences) and subjected to density gradient centrifugation using 40% and 70% Percoll solutions (GE Healthcare). Mesenteric lymph nodes were excised and single cell suspensions were prepared by mechanical disruption for T-cell isolation. For flow cytometry, single cell suspensions were incubated with Fc Block for 10 minutes, and then stained with antibodies and Fc Block for 20 minutes at 4 °C. Dead cells were excluded using either a Live/Dead Fixable Cell Stain Kit (ThermoFisher Scientific) or DAPI (Sigma). Intracellular proteins were stained using either the BD Biosciences Fixation/Permeabilization Solution Kit or the eBioscience Transcription Factor staining kit.

Cells were run on a FACSCanto II instrument (BD Biosciences) and data were analyzed using FlowJo (FlowJo LLC). Cells counts were performed with counting beads (eBioscience).

For flow cytometry, the following fluorophore-labeled monoclonal antibodies were used: (i) CD90.2 (53-2.1), GATA3 (L50-823), CD196 (140706), and CD4 (RM4-5) from BD Biosciences; (ii) CD8α (53-6.7), CD62L (MEL-14), TCRγδ (ebioGL3), RORγ(t) (AFKJS-9), Eomes (Dan11mag), CD45 (30-F11), Foxp3 (FJK-16 s), and CD44 (IM7) from eBioscience; and (iii) CD8β (YTS156.7.7),CD3ε (145-2C11), CD19 (6D5), I-A/I-E (M5/114.15.2),CD11c (N418), CD11b (M1/70), CD103 (2E7), and CD335 (29A1.4) from Biolegend.

Lymphocytes from colonized mice were stimulated *ex vivo* either with 10 ng/mL of IL-23 and IL-1β for 3.5 hours to measure IL-17A and IL-22 production or with 0.1 nM phorbol 12-myristate 13-acetate (PMA; Sigma-Aldrich) and 1 μg/mL ionomycin (Sigma-Aldrich) for 4 hours to measure IL17A and IFN-γ production. In experiments requiring intracellular cytokine staining, Golgi Plug (Becton Dickinson) was present for the last 3 hours of culture. Surface staining was performed, followed by fixation and permeabilization (Cytofix/Cytoperm Plus kit; Becton Dickinson). Intracellular cytokine contents were determined using the mAbs IL-17A (eBio17B7; eBioscience), IFN-γ (XMG1.2; BD) and IL-22 (Poly5164; eBiolegend).

Assaying spleens for viable bacteria

Nine days after the first gavage, spleens were harvested at the time of euthanasia and homogenized in 1.0 mL PBS. 100 μL of the resulting homogenate was plated on BHI horse blood agar and incubated for at 37 ºC for 12-16 hours under aerobic conditions. Cultured isolates were initially classified by their morphotypes, followed by Vitek MALDI-TOF mass spectrometry and sequencing full-length 16S rDNA amplicons (generated from their genomic DNA using primers 8F and 1391R).

STATISTICAL ANALYSIS

Effects of nutritional intervention on ponderal and linear growth

A paired t-test between WAZ and LAZ at the time of endoscopy and WAZ and LAZ just prior to beginning the nutritional intervention was performed to determine the effects of the nutritional intervention on ponderal/linear growth.

Relationship between histopathologic score and linear growth

The effect of histopathologic severity on LAZ was assessed by creating a linear model containing histopathologic score, sex, and age at the time of endoscopy as covariates and LAZ at the time of endoscopy as the outcome; the coefficient reported in **Table 1** describes the relationship between histopathologic score and LAZ, controlling for sex and age.

Relationship between fecal biomarkers and linear growth

Concentrations of fecal biomarkers measured from the 110 stunted children who failed nutritional intervention were log-transformed and correlated against LAZ.

Relationship between a fecal 'EE biomarker score' and LAZ

A previously described environmental enteropathy score ('EE score') based on trichotomized measurements of fecal alpha-1-antitrypsin (AAT), myeloperoxidase (MPO), and neopterin (NEO) was adapted for this study (21). Briefly, the published 'EE score' is calculated by the following equation:

$$EE\ score = 2 \times (AAT\ category) + 2 \times (MPO\ category) + (NEO\ category)$$

$$category = 0 \quad \leq 25th\ percentile$$

$$category = 1 \quad 25th - 75th\ percentile$$

$$category = 2 \quad \geq 75th\ percentile$$

The total 'EE score' ranges from 0-10, with 10 being the most severe. However, because the score is based on quantiles, comparisons between patient populations are impacted by the unique distribution of biomarker levels in different study cohorts. We therefore adapted the 'EE score' into a continuous composite variable as described in (22) and defined by the following equation:

$$EE\ biomarker\ score = 2 \times (AAT\ in\ mg/g) + 0.2 \times (MPO\ in\ \mu g/mL) + (NEO\ in\ \mu mol/L)$$

This modified score is based on the absolute concentrations of each fecal biomarker, preserves the relative contribution of each biomarker to the total 'EE biomarker score', and brings the score into a comparable

arithmetical range to the originally published ‘EE score’. [In the cohort of 110 stunted children who did not respond to nutritional intervention, the trichotomized ‘EE score’ and the continuous ‘EE biomarker score’ were well correlated (Pearson rho=0.64, p=5.56x10^{-14})]. The resulting ‘EE biomarker score’ was log-transformed and correlated against LAZ.

Comparative analyses of the plasma proteomes of healthy children and children with EED

Three comparisons using an empirical Bayesian linear modeling framework [*limma* (2)] were performed: healthy vs. BEED pre-intervention; healthy vs. BEED post-intervention; and BEED pre- vs. post-intervention. For the former two analyses, linear models including ‘group’ (healthy or BEED), age and sex as covariates and the abundance of a protein as the response were generated using *limma*. For the latter analysis, ‘group’ was the primary predictor and patient ID was added as a covariate to encode a paired analysis in *limma*. For each of the three analyses, only individuals with a duodenal biopsy and a histopathologic diagnosis of EED were included (n=80).

Relating fecal biomarkers and plasma proteins to LAZ

To determine the relationship between plasma proteins and LAZ at the time of endoscopy, Pearson correlation coefficients were calculated between each of the 4077 log2-transformed, quantile-normalized plasma protein abundances and LAZ. To identify modifiers of the effects of IGF-1 on LAZ, a separate linear model was created for each plasma protein or log-transformed fecal biomarker using the following formula:

$$LAZ\ at\ Endoscopy = \alpha_0 + \alpha_1(IGF1) + \alpha_2(Protein) + \beta(IGF1 \times Protein) + \varepsilon$$

where α_0 is the model intercept, α_1 and α_2 are the coefficients for the main effects of IGF1 and the putative interacting protein, respectively, β is the coefficient for the interaction term, and ε is the residual. Statistical significance was calculated using the ‘lm’ function in R, and is reported in **Table S4** for the interaction coefficient β. Linear models for which coefficients are reported were generated after z-scoring the independent variables. Thus, the β coefficient represents the change in the effect of IGF-1 on LAZ after a unit standard deviation change in the interacting protein.

Identifying modules of co-expressed duodenal proteins

Independent components analysis (ICA) was initially developed in the field of signal processing to solve the blind-source separation (BSS) problem (23). One formulation of the BSS problem is trying to identify which instruments are playing in an orchestra while only hearing the entire orchestra play at the same time. More recently, ICA was shown to be an effective module detection method to identify biologically meaningful co-expression profiles in various types of expression data when formulated as a BSS problem (24). Given an observed signal (protein expression), ICA attempts to deconvolve the signal into statistically independent components (ICs). These ICs represent modules of proteins whose members have distinct expression profiles compared to proteins in other ICs/modules. The measured variation in protein expression between samples can thus be thought of as alterations in the activation or inhibition of statistically orthogonal biological pathways that have been 'mixed' together; the resulting admixture is the plasma proteomic data measured by SomaScan, while the discovered ICs represent pathways of potential interest.

The number of ICs was determined using a random matrix theory approximation described elsewhere (25). ICA was performed using the R implementation of the JADE algorithm (23), which attempts to maximize the difference in fourth-order moments as a proxy for identifying statistically orthogonal ICs. For the n x m protein expression matrix (n = number of proteins, m = number of subjects), ICA returns a n x s protein projection matrix where each element is the projection of protein n along IC/module s, as well as a m x s subject projection matrix where each element is the projection of patient m along IC/module s. Modules were defined by identifying proteins with statistically significant (FDR-corrected p-value < 0.05) projections along a given IC as calculated by the 'fdrtool' package in R (26).

To determine the functional significance of modules identified by ICA, we performed GO overrepresentation analysis on each module using the R package *topGO* (29). *TopGO* attempts to de-correlate dependencies between GO terms when calculating enrichment scores using a combination of gene (protein) removal and maximizing enrichment scores within a given neighborhood on the GO directed acyclic graph. The *weight01* method was used to calculate enrichment, and a p-value < 0.05 was

used to define statistically significant enrichment of a GO term. The p-value represents the adjusted p-value calculated by *topGO*, which applies a Bonferroni-like correction during p-value calculations. All GO 'biological processes' with 10 or more proteins represented in the 2619 duodenal proteins passing QC were included in the analysis.

Cross-correlation singular value decomposition (CC-SVD) analysis

CC-SVD decomposes a cross-correlation matrix into flexible modules of features with conserved correlation profiles. Beginning with two matrices with dimension *m x n* and *m x p*, CC-SVD computes a Pearson cross-correlation matrix (*n x p*). Next, the matrix is decomposed using singular value decomposition (SVD) into left and right singular matrices that contain the left and right singular vectors, also known as eigenvectors (EVs); these EVs represent correlation profiles, or modules of highly cross-correlated features.

The number of modules was determined using the random matrix approximation described elsewhere (25). The module size was chosen to include duodenal proteins with the top 50 most positive and most negative projections. All 14 ASVs were included. Module members were then extracted from the cross-correlation matrix and plotted using the 'corrplot' function in R (28) in rank order by their projections onto the EV, with larger magnitude projection values indicating a stronger correlation profile. Proteins with positive projections are positively correlated with ASVs with positive projections, and negatively correlated with ASVs with negative projections. Conversely, proteins with negative projections are positively correlated with ASVs with negative projections, and negatively correlated with proteins with positive projections. Thus, the approach leverages the ability of SVD to identify orthogonal variance in a cross-correlation matrix in order to find modules of covarying features across multiple feature types; this approach was used to assess relationships between the duodenal proteome and the absolute abundances of duodenal bacterial taxa.

CC-SVD was also performed on the 4077 plasma proteins and the top 100 (50 positively and 50 negatively correlated) duodenal microbiota-associated duodenal proteins to identify members of the plasma proteome that significantly correlated with the profile of the 100 duodenal proteins. Projections of

each plasma protein along the first eigenvector, which represent the strength of association between the plasma protein and the ensemble of 100 duodenal proteins, were z-scored and converted into p-values under the assumption that the projections were distributed according to a normal distribution of $\mu = 0$ and $\sigma^2 = 1$. FDR correction was applied to these empirical p-values using the Benjamini-Hochberg method.

Analysis of the representation of duodenal 'core' taxa in fecal samples from healthy children or children with EED

Statistically significant differences in the relative abundances of 'core' group duodenal taxa in the fecal microbiota of healthy children or children with EED were individually tested using a two-sided Mann-Whitney U test (FDR-corrected $p<0.05$). To determine whether the ensemble of these 14 taxa were significantly different in fecal samples collected from the two groups of children, PCA was performed on the DESeq2-normalized and transformed counts (see above), Euclidian distances between each fecal sample were calculated on the resulting principal components, and permutation ANOVA (99999 permutations) implemented using the 'adonis2' algorithm from the *vegan* package was used to calculate whether the 14 duodenal taxa could separate healthy children from children with EED (29,30).

DATA DEPOSITION

V4-16S rDNA sequences in raw format prior to post-processing and data analysis, the genome sequences of cultured duodenal bacterial strains, shotgun sequencing data produced from aspirate DNA recovered from intestinal luminal contents harvested from gnotobiotic mice and duodenal RNA-Seq datasets generated from these animals have been deposited at the European Nucleotide Archive under study accession PRJEB32184. Software developed for the analyses is available at GitHub.

SUPPLEMENTARY RESULTS

Comparison of the plasma proteomes of children with EED prior to and after nutritional intervention and of their healthy counterparts

To obtain a molecular definition of the biological state of the 80 children with histopathologic evidence of

EED, we compared the abundances of 4077 proteins, including biomarkers and mediators of diverse physiologic, metabolic, immune, neurodevelopmental and other host functions, in plasma samples collected just prior to the nutritional intervention with their abundances in plasma samples collected from age-matched children (n=21) living in Mirpur who had healthy growth phenotypes (*limma* unadjusted p<0.01, **Figure S2, Table S5A,B**). Soluble growth hormone (GH) receptor (also known as GH binding protein) which regulates GH activity (31), leptin, insulin-like growth factor acid-labile subunit (IGFALS) which increases IGF-1 half-life (32), and soluble klotho, a master regulator of serum phosphate levels that also induces adipocyte differentiation and modulates sensitivity to IGF-1 (33), were all lower in stunted compared to healthy children.

We compared the plasma proteomes of healthy children to those of children with EED at the time of biopsy when the nutritional intervention had been completed. Abundances of GH, leptin, and klotho remained depressed in EED children even after nutritional intervention. Proteins elevated in the plasma of children with EED included the pro-inflammatory chemokine CXCL11, platelet glycoprotein V (GP5) which mediates platelet adhesion and is a marker for thrombosis (34), the collagenase matrix metalloproteinase 1 (MMP1), whose expression is elevated during mucosal injury in patients with inflammatory bowel disease (35), and pappalysin-1 (PAPPA), a protease that regulates IGF-1 bioavailability by cleaving IGF binding proteins (32) (*limma* unadjusted p<0.01, **Figure S2**, **Table S5C,D**)

Comparing the plasma proteomes of the 80 children collected before and after the nutritional intervention revealed 69 proteins that were elevated after treatment; these proteins are involved in diverse functions, including growth [IGF-1, thyroid stimulating hormone, procollagen C-endopeptidase enhancer 2 (PCOLCE2)], digestion [beta-Ala-His-dipeptidase, amylases] and neurobiology [neuropeptide S (NPS), slit homolog 2 (SLIT2), SLIT and NTRK-like protein 3 (SLITRK3)]. Levels of 39 plasma proteins were significantly reduced after treatment including stanninocalcin-1, an inhibitor of the metalloprotease pappalysin-1 that cleaves IGF binding proteins (IGFBPs) to modulate the activity of IGF-1, and growth-differentiation factor 15 which is elevated in children with severe acute malnutrition and implicated in

anorexia and muscle wasting (36) (*limma* unadjusted p<0.01; see **Figure S2** and **Table S2E,F**).

Characterization of immune cell populations in gnotobiotic mice colonized with the EED donor-derived bacterial consortium

To characterize immune cell populations in the small intestinal epithelium and lamina propria plus mesenteric lymph nodes, two follow-up experiments were performed where the entire length of the small intestine and mesenteric lymph nodes were recovered from mice euthanized 7 days following the final gavage with (i) the full 39-member bacterial consortium or (ii) the cecal contents of a conventionally-raised mouse (n=5 animals/treatment group/experiment; **Table S12**). Within the epithelial compartment, there were no statistically significant differences in the absolute numbers or frequencies of TCRαβ or TCRγδ lymphocytes between the two groups of animals. In the small intestinal lamina propria compartment, we observed a significant increase in the number and frequency of CD4+ T-cells and among them Rorγt+ Th17 but not Foxp3+ Treg cells in mice colonized with the EED consortium. Moreover, *ex vivo* tests of effector function in small intestinal lamina propria-derived activated T cells disclosed increased IL17A production but no significant difference in IFNγ production in mice harboring the EED community compared to the CONV-D group.

The increase in Th17 cells in EED mice was paralleled by changes in the innate immune compartment, particularly among ILC3 subsets which, along with Th17 cells, contribute to the maintenance of the intestinal epithelial barrier through secretion of IL-17 and IL-22A (37). CCR6+ ILC3s, which are the major innate source of IL-17, were significantly decreased, whereas NKp46+ ILC3s, which predominantly produce IL-22, were significantly increased in the small intestinal lamina propria of EED consortium-colonized mice **(Table S12**). *Ex vivo* stimulation experiments disclosed that IL-17A production by CCR6+ ILC3s was also significantly reduced in mice harboring the bacterial consortium, while no significant differences were observed in IL-22 production by NKp46+ ILC3s between the two groups of animals (**Table S12**). Thus, the observed increase of Th17 cells may reflect a compensatory response to a defect in CCR6+ ILC3s.

Mice colonized with the EED consortium also had significantly increased numbers of lamina propria CD11b+ CD103+ dendritic cells and Th17 cells among the memory CD4+ T cell ($CD44^{hi}CD62L^{lo}$) population present in their mesenteric lymph nodes (**Table S12**). Dendritic cells present in intestinal tissues acquire antigens and migrate to mesenteric draining lymph nodes where they prime antigen-specific T cells. Interestingly, CD4+ T-cells producing IL-17A are almost exclusively primed by CD11b+CD103+ dendritic cells in mesenteric lymph nodes (38-40), supporting our observation that colonization with the duodenal bacterial consortium from children with EED induces an immune response in the small intestine biased towards the generation of Rorγt+Th17 cells. Such a response, if sustained, may contribute to intestinal inflammation.

SUPPLEMENTARY FIGURES

Figure S1 – Scoring system for grading severity of histopathologic changes in duodenal biopsies.

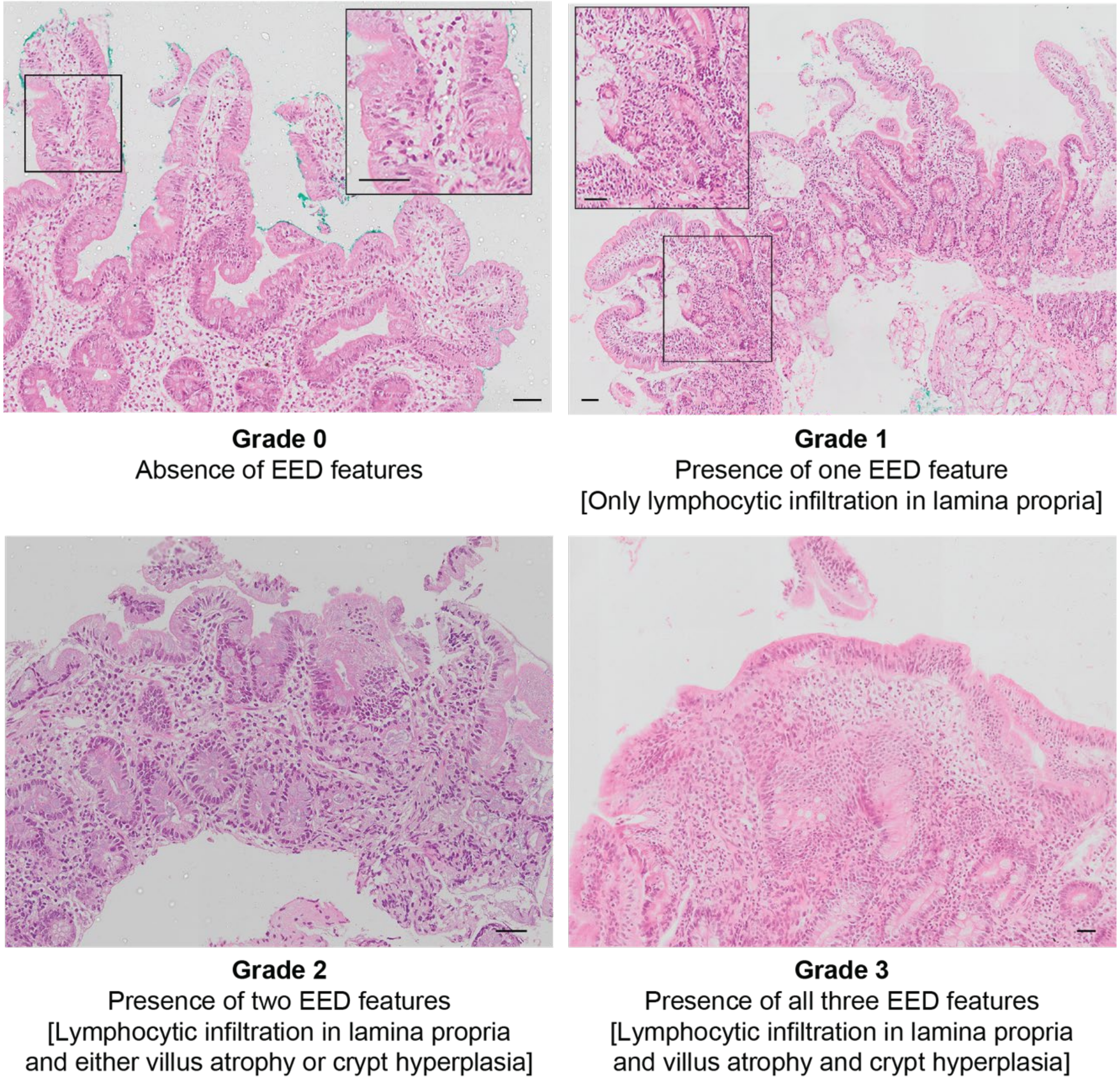

Photomicrographs illustrate the features of biopsies assigned to different levels of disease severity. The insets show higher power views of the boxed areas. Scale bars, 25 μm

Figure S2 –Plasma proteins whose abundances are significantly different between children living in Mirpur with EED, prior to and after their nutritional intervention, and children from Mirpur judged to have healthy growth phenotypes. Shown is an 'UpSet' plot generated in R using data shown in **Table S5.**

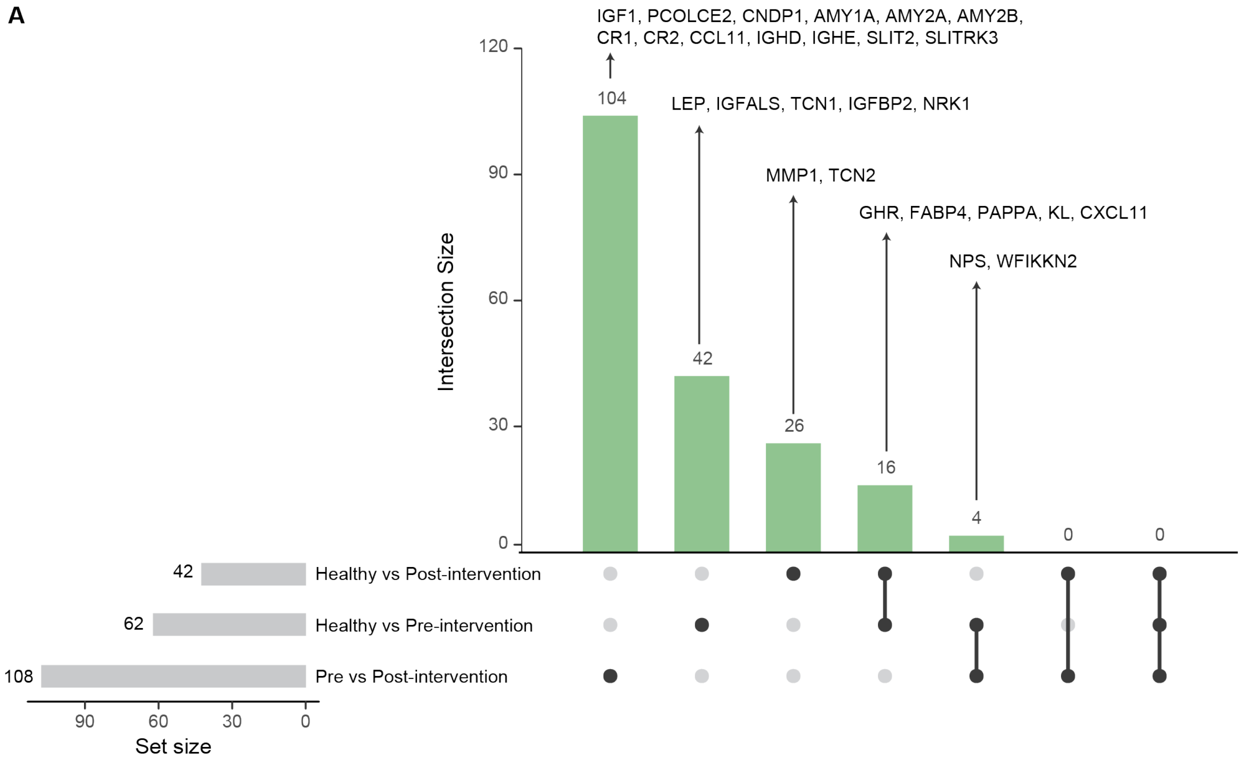

B

Symbol	Name	Function
AMY1A	Amylase alpha 1A	Digestive enzyme
AMY2A	Amylase alpha 2A	Digestive enzyme
AMY2B	Amylase alpha 2B	Digestive enzyme
CCL11	Eotaxin-1	Canonical eosinophil chemokine
CNDP1	Beta-ala-his dipeptidase	Metallopeptidase associated with obesity risk
CR1	Complement receptor 1	Immune receptor regulating complement activation
CR2	Complement receptor 2	B-cell complement receptor
CXCL11	C-X-C motif chemokine ligand 11	T-cell chemokine
FABP4	Adipocyte fatty acid binding protein	Adipokine
GHR	Growth hormone receptor	Canonical receptor for growth hormone
IGF1	Insulin-like growth factor 1	Canonical mediator of linear growth
IGFALS	IGF acid-labile subunit	IGF-1 stabilizing protein
IGFBP2	IGF binding protein 2	Inhibitory IGF-1 binding protein
IGHD	Immunoglobulin D	Immunoglobulin produced by immature B-cells
IGHE	Immunoglobulin E	Mast cell-activating immunoglobulin produced by plasma cells
KL	Klotho	Serum phosphate regulation, immune modulation, aging
LEP	Leptin	Canonical adipokine
MMP1	Matrix metalloproteinase 1	Collagenase implicated in bone resorption and intestinal inflammation
NPS	Neuropeptide S	Suppression of anxiety and appetite
NRK1	Nicotinamide riboside kinase 1	NAD metabolism
PAPPA	Pappalysin-1	Enzyme liberating IGF-1 from inhibitory binding proteins
PCOLCE2	Pro-collagen c-endopeptidase enhancer 2	Cartilage remodeling
SLIT2	Slit guidance ligand 2	Axon guidance; glucose homeostasis
SLITRK3	SLIT and NTRK like family member 3	Synaptic development
TCN1	Transcobalamin-1	Prevents degradation of Vitamin B12
TCN2	Holo-transcobalamin-2	Prevents degradation of Vitamin B12
WFIKKN2	Growth and differentiation factor associated serum protein 1	Inhibitor of the GDF family growth factors

(A) Three sets of differentially abundant proteins were included in this analysis: healthy vs post-intervention, healthy vs pre-intervention, and pre- vs post-intervention. Each vertical green bar represents

the number of proteins in a given intersection between sets. The sets being queried are denoted by the black circles below the bar graph. A single dot indicates proteins unique to a set, while multiple dots represent proteins that are shared (intersected) by the sets. The horizontal gray bars on the left indicate the total number of differentially abundant proteins in the set. Select proteins from each set intersection are labelled. **(B) A**nnotations for proteins listed in panel A.

Figure S3A – CC-SVD analysis of duodenal proteins and duodenal bacterial taxa

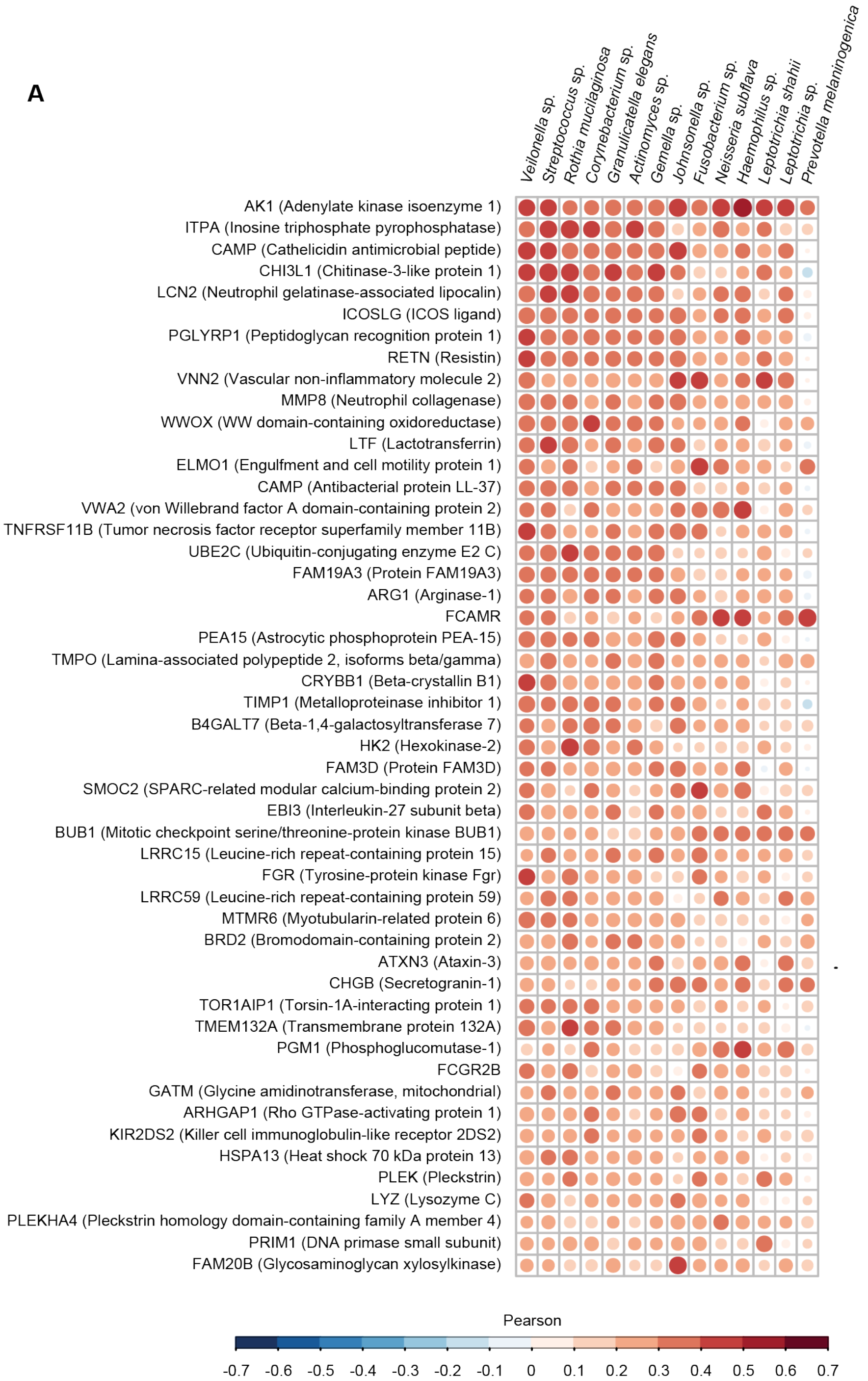

(A) The 50 most positively correlated duodenal proteins with the absolute abundances of 14 core duodenal bacterial taxa identified by CC-SVD. Each circle represents the Pearson rho between the abundance of a duodenal protein and duodenal bacterial taxa. The size and shading of a circle represent

the strength of the correlation (larger and darker circles represent stronger correlations). Positive correlations are in red.

Figure S3B – CC-SVD analysis of duodenal proteins and duodenal bacterial taxa

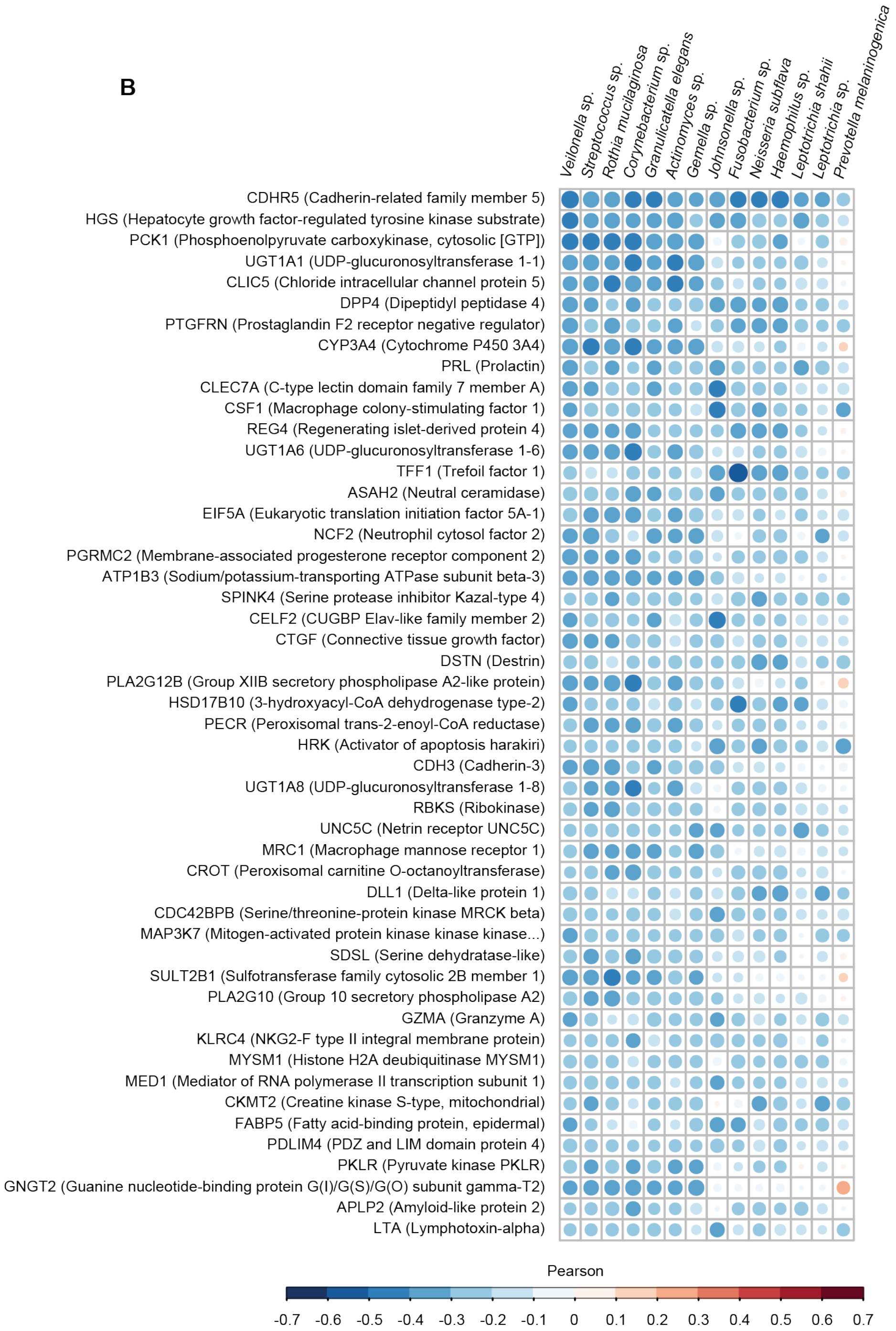

(B) The 50 most negatively correlated proteins with the absolute abundances of 14 core duodenal bacterial taxa identified by CC-SVD. Each circle represents the Pearson rho between the abundance of a duodenal protein and duodenal bacterial taxa. The size and shading of the circle represent the strength of

the correlation (larger and darker circles represent stronger correlations). Negative correlations are in blue.

Figure S4 – Correlations between plasma LCN-2 or PTS and duodenal proteins

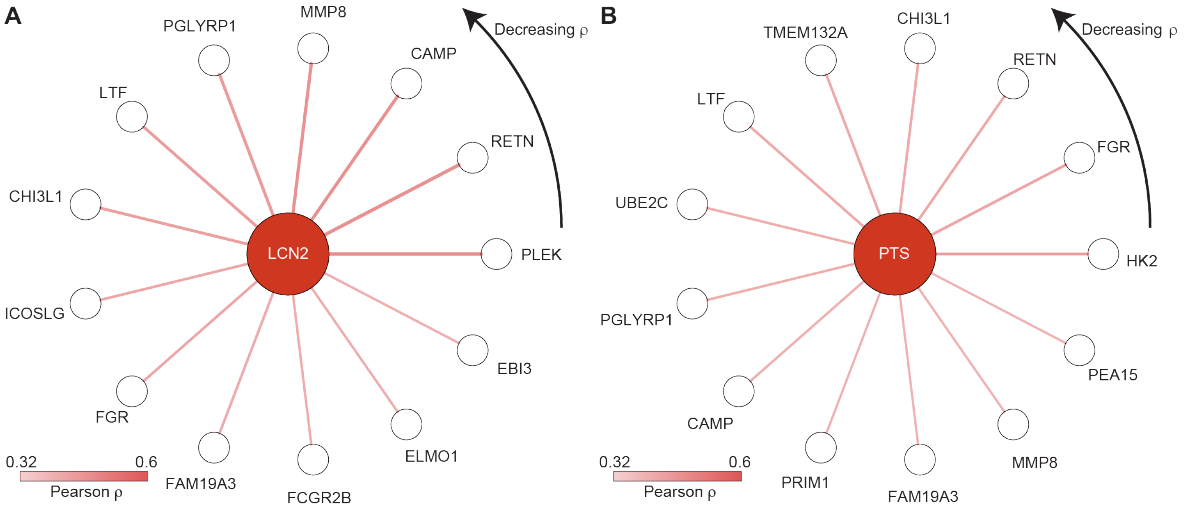

C

Symbol	Name	Function
CAMP	Cathelicidin antimicrobial peptide	Innate immune defense against bacteria
CHI3L1	Chitinase-3-like protein 1	Biomarker of inflammation in multiple disease contexts
EBI3	Interleukin-27 Beta	Beta-subunit of IL-27, regulates T-cell inflammatory responses
ELMO1	Engulfment and cell motility 1	Inflammatory mediator of neutrophil activation and phagocytosis
FAM19A3	TAFA chemokine like family member 3	Chemokine-related protein
FCGR2B	Fc gamma receptor II-B	Regulation of immunoglobulin-dependent phagocytosis
FCGR2B	Fc Fragment of IgG IIb	Immunoglobulin Fc receptor involved in phagocytosis of immune complexes
FGR	Src-family kinase Fgr	Proliferative kinase involved in immune regulation
HK2	Hexokinase 2	Glucose metabolism; regulation of monocyte differentiation
ICOSLG	Inducible costimulator ligand	Immune ligand involved in wound healing
LTF	Lactotransferrin	Antimicrobial peptide; mucosal defense
MMP8	Matrix metalloproteinase 8	Neutrophil collagenase upregulated in intestinal inflammation
PEA15	Proliferation and apoptosis adapter protein 15	Phosphoportein highly expressed in colorectal cancer and liver metastases
PGLYRP1	Peptidoglycan recognition protein 1	Bactericidal proinflammatory cytokine
PLEK	Pleckstrin	Platelet protein
PRIM1	DNA primase subunit 1	Component of the DNA primase involved in DNA replication
RETN	Resistin	Proinflammatory cytokine; induces insulin resistance
TMEM132A	Transmembrane protein 132A	Transmembrane protein involved in nervous system development
UBE2C	E2 Ubiquitin-conjugating enzyme C	Autophagy repressor overexpressed in gastric cancers and other neoplasias

(**A,B**) Star networks of correlations between plasma lipocalin-2 (LCN-2) and 6-pyruvoyltetrahydropterin synthase (PTS) and core taxa-associated duodenal proteins. As indicated by the color key, edge transparency/color corresponds to correlation strength (darker edges denote a stronger correlation). The duodenal proteins with the strongest correlations to LCN-2 and PTS are indicated by the tail of the arrowhead at 3 o'clock; duodenal proteins with progressively weaker correlations are distributed in a counter-clockwise fashion from this position. (**C**) HUGO gene symbols and functions of their protein products.

Figure S5 – Relative abundances of core duodenal taxa in the fecal microbiota of children living in Mirpur who have healthy growth phenotypes (n=27) and those with EED (n=48).

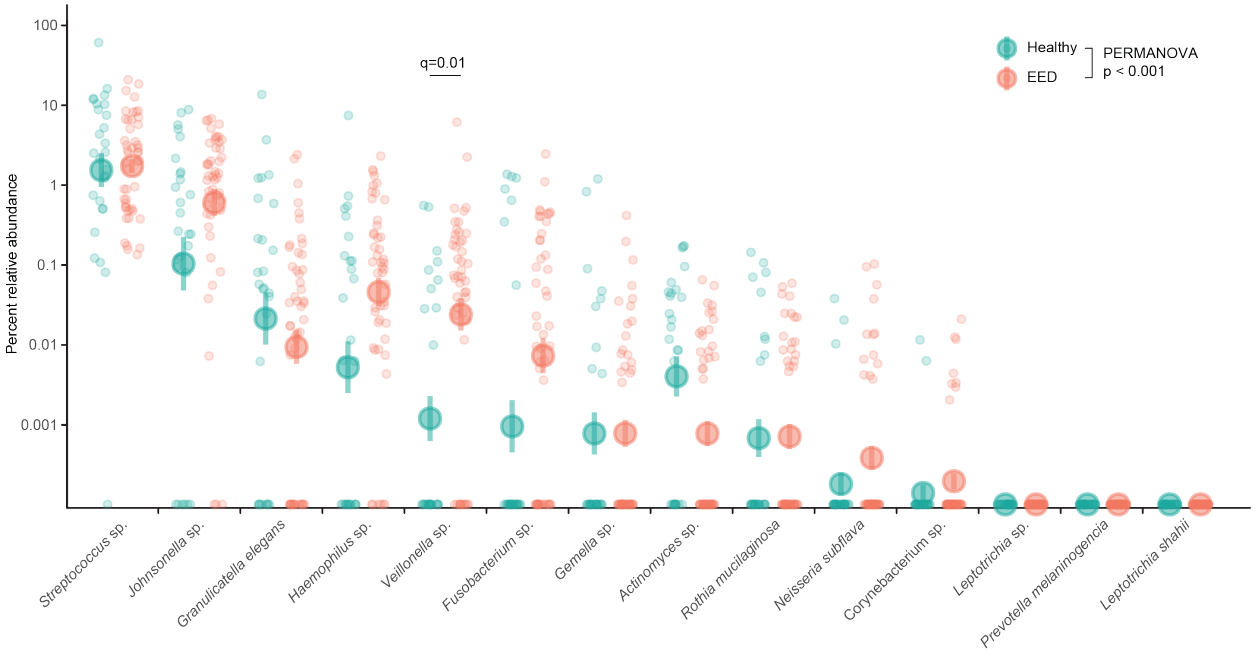

The large circle represents the mean and the vertical line denotes the standard error. Differential abundance analysis for each of the 14 duodenal 'core taxa' was performed using a two-sided Mann-Whitney U test (FDR-corrected). PCA of normalized, variance-stabilized counts followed by permutation ANOVA (PERMANOVA) was used to test for significant separation between healthy versus EED fecal samples.

Figure S6 – Binary phenotype matrix of *in silico* predictions of metabolic functions of 39 cultured bacterial strains recovered from BEED duodenal aspirates.

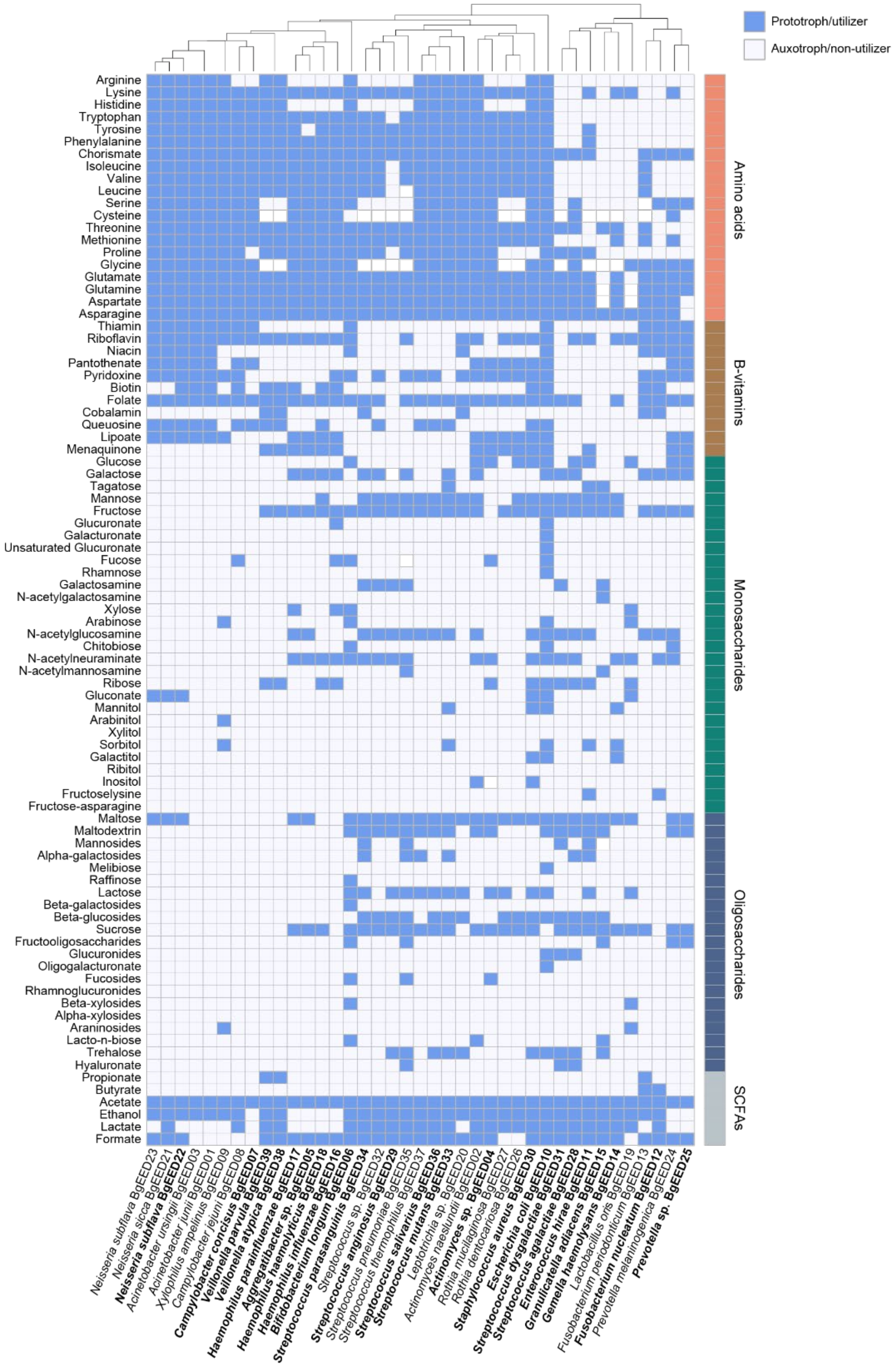

In this binary phenotype matrix "1" denotes a predicted prototroph and "0" a predicted auxotroph as inferred from *in silico* reconstruction of the respective mcSEED metabolic pathways/modules for amino

acid and B-vitamin biosynthesis. In the case of carbohydrates, '1' and '0' refer to a strain's predicted ability or inability, respectively, to utilize the indicated mono-, di-, or oligosaccharide. Binary phenotypes for fermentation end products (short chain fatty acids, SCFA) are related to a strain's predicted ability to produce the indicated compound. In bold are the 23 strains detected at greater than 0.01% relative abundance at one or more locations along the gastrointestinal tract, averaged across all recipient mice.

Figure S7 – Analysis of the distribution of bacterial strains along the length of the intestines of gnotobiotic mice.

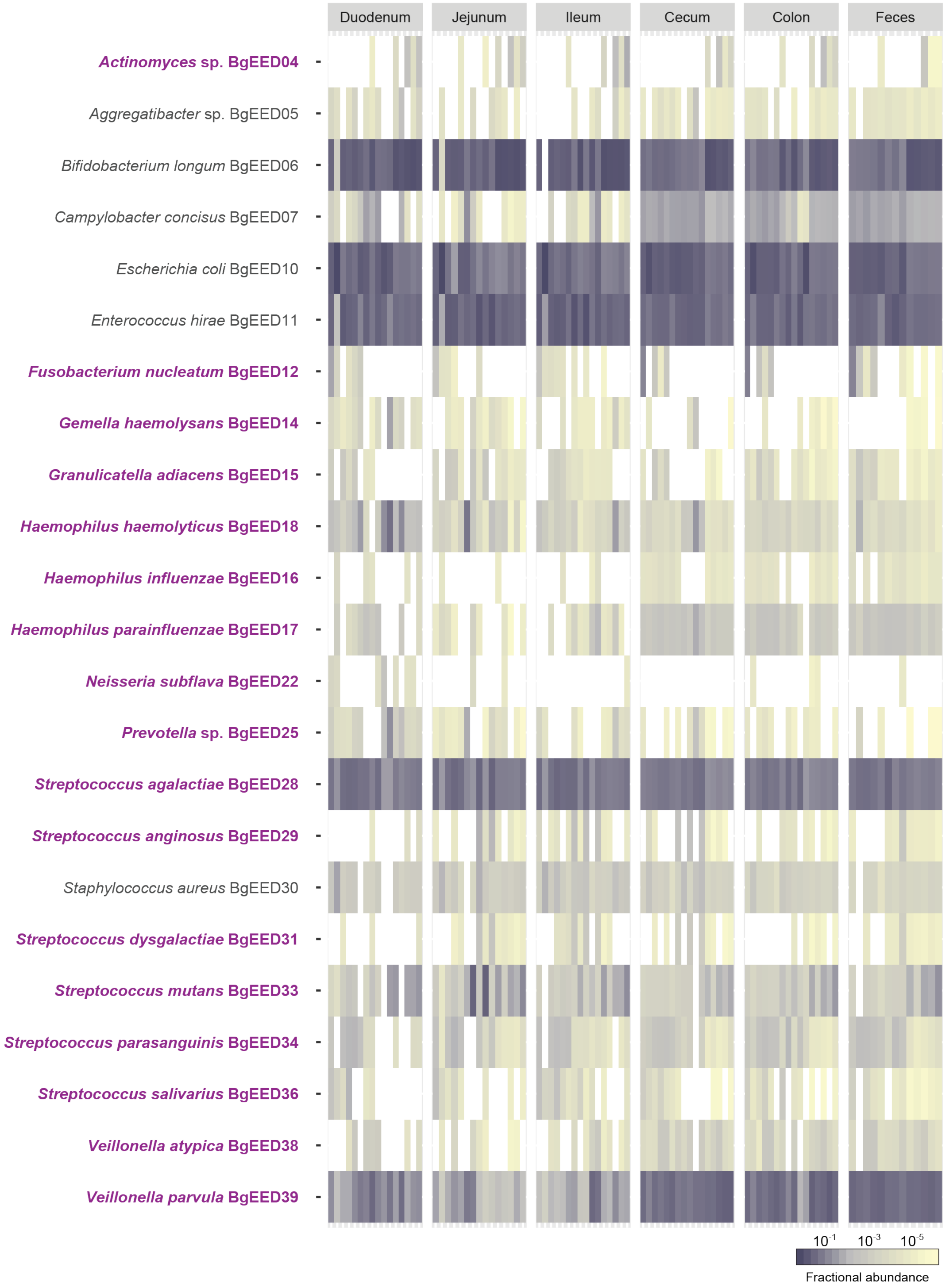

Strains in the 39-member cultured community belonging to the same genus as the 14 'core group' duodenal taxa are bolded and purple.

SUPPLEMENTARY TABLES

Table S1 – Biospecimens analyzed from BEED participants and from healthy controls

Table S2 – Clinical characteristics of subgroups in the BEED study. Values represent: mean ± standard deviation; number (percentage); mean difference (95% CI); median [interquartile range]. WAMI index refers to *Water-sanitation-hygiene, Asset status, Maternal education status, and monthly Income*. Statistically significant differences in characteristics between groups were performed using an unpaired t-test unless otherwise noted.

*Statistically significant improvement in anthropometric measure as determined by paired t-test ($p<0.05$).

**Statistically significant improvement in anthropometric measure as determined by paired t-test ($p<0.01$)

***Statistically significant improvement in anthropometric measure as determined by paired t-test ($p<0.001$)

† Statistical significance determined using a Chi-squared test.

‡ Statistical significance determined using a Mann-Whitney U test.

Table S3 – Correlations between plasma or duodenal proteins and LAZ. (A) Correlations between plasma proteins and LAZ. **(B)** Correlations between duodenal proteins and LAZ. Correlations with an unadjusted $p<0.05$ are listed. Correlations were calculated for n=80 children with EED.

Table S4 – Results of linear modeling of interactions between IGF-1 and plasma proteins/fecal biomarkers on LAZ. The β coefficient and standard error describe the effects of one standard deviation change of the interacting protein on the relationship between IGF-1 and LAZ. Both IGF-1 and the

interacting protein were included in the model as main effects. Proteins with an unadjusted $p<0.05$ interaction coefficient are listed.

Table S5 – Analysis of the plasma proteomes of children living in Mirpur who had healthy growth or BEED children who were stunted or at risk for stunting and failed nutritional intervention. Differential abundance analysis (*limma*). 'logFC' indicates the log2-fold-change of a given protein in the test group compared to the reference group. **(A)** Proteins more abundant in the plasma of healthy children compared to plasma from BEED children prior to nutritional intervention. **(B)** Proteins more abundant in plasma from BEED children prior to nutritional intervention compared to plasma from healthy children. **(C)** Proteins more abundant in the plasma of healthy children compared to plasma from BEED children after completing nutritional intervention. **(D)** Proteins more abundant in plasma from BEED children after completing nutritional intervention compared to plasma from healthy children. **(E)** Proteins more abundant in the plasma of BEED children after completing nutritional intervention compared to prior to nutritional intervention. (**F**) Proteins more abundant in the plasma of BEED children prior to nutritional intervention compared to after completing nutritional intervention.

Table S6 – Relative abundances of bacteria taxa (ASVs) and total bacterial load in duodenal aspirates from BEED children. Bacterial load is the ratio of the summed relative abundance of all taxa that are not classified as the spike-in bacteria *A. acidophilus* compared to the relative abundance of the *A. acidophilus* spike-in.

Table S7 – Duodenal proteins and assigned GO Biological Process terms in Module 1. (A) Proteins belonging to duodenal Module 1 and their associated module membership, defined as their projection along the independent component describing the module. **(B)** GO Biological Processes that are overrepresented by the proteins belonging to module 1 as determined by *topGO* (adjusted $q< 0.05$).

Table S8 – qPCR assays for enteropathogens. Enteropathogen burden in **(A)** duodenal aspirates from children with EED (n=36) and **(B)** feces from children with EED (n=23) and healthy reference children (n=19). Reported values are absolute measurements of DNA mass in pg (all bacteria and protists) and copy number (all RNA viruses).

Table S9 – Mirpur-18 diet given to gnotobiotic mice. (A) Ingredients used to formulate diet. **(B)** Nutritional analysis of diet.

Table S10 – Distribution of members of the bacterial consortium along the length of the gastrointestinal tracts of recipient gnotobiotic mice. Values represent fractional abundances. Rows with '-' indicate missing samples.

Table S11 – Differential expression of genes in the duodenums of gnotobiotic mice colonized with the cultured bacterial consortium from children with EED versus CONV-D controls. List of differentially expressed genes ranked according to p-value (DESeq2).

Table S12 – Flow cytometric analysis of immune cell populations present in the small intestinal epithelium and lamina propria plus mesenteric lymph nodes of gnotobiotic mice colonized with the EED bacterial consortium and CONV-D controls.

ONLINE SUPPLEMENTARY MATERIALS

Online Supplementary Data Table 1 - Duodenal proteome module membership in children with EED and GO Biological Process enrichment for each module.

Online Supplementary Data Table 2 – Duodenal aspirate microbial community composition of children with EED determined using MEtaPhlAn2.

Online Supplementary Data Table 3 – Annotation of the genomes of 39 bacterial strains cultured from duodenal aspirates obtained from Mirpur children with EED.

SUPPLEMENTARY REFERENCES

1. Mahfuz M, Das S, Mazumder RN, et al. Bangladesh Environmental Enteric Dysfunction (BEED) study: Protocol for a community-based intervention study to validate non-invasive biomarkers of environmental enteric dysfunction. BMJ Open 2017;7:e017768.
2. Ritchie ME, Phipson B, Wu D, et al. Limma powers differential expression analyses for RNA-sequencing and microarray studies. Nucleic Acids Res 2015;43:e47.
3. Blanton LV, Charbonneau MR, Salih T, et al. Gut bacteria that prevent growth impairments transmitted by microbiota from malnourished children. Science 2016;351: aad3311.
4. Callahan BJ, McMurdie PJ, Rosen MJ, Han AW, Johnson AJA, Holmes SP. DADA2: High rsolution sample inference from Illumina amplicon data. Nat Methods 2016;13:581-83.
5. McMurdie PJ, Holmes S. Phyloseq: An R Package for Reproducible Interactive Analysis and Graphics of Microbiome Census Data. PLoS One 2013;8:e61217.
6. Stämmler F, Glasner J, Hiergeist A, et al. Adjusting microbiome profiles for differences in microbial load by spike-in bacteria. Microbiome 2016;4:28.
7. Anders A, Huber W. Differential expression analysis for sequence count data. Genome Biol 2010;11:R106.
8. Truong DT, Franzosa EA, Tickle TL, et al. MetaPhlAn2 for enhanced metagenomic taxonomic profiling. Nat Methods 2015;12:902-03.
9. Liu J, Grats J, Amour C, et al. A laboratory-developed taqman array card for simultaneous detection of 19 enteropathogens. J Clin Microbiol 2013;51:472–80.

10. Bankevich A, Nurk S, Antipov D, et al. SPAdes: A New Genome Assembly Algorithm and Its Application to Single-Cell Sequencing. J Comput Biol 2012;5:455-77.

11. Seemann T. Prokka: Rapid prokaryotic genome annotation. Bioinformatics 2014;14:2068-69.

12. Overbeek R, Olson R, Pusch GD, et al. The SEED and the Rapid Annotation of microbial genomes using Subsystems Technology (RAST). Nucleic Acids Res 2014;42:D206-14.

13. Parks DH, Imelfort M, Skennerton CT, et al. CheckM: assessing the quality of microbial genomes recovered from isolates, single cells, and metagenomes. Genome Res 2015;7:1043-105.

14. Rodionov DA. Comparative Genomic Reconstruction of Transcriptional Regulatory Networks in Bacteria. Chem Rev 2007;8:33467-97.

15. Overbeek R, Bartels D, Vonstein V, Meyer F. Annotation of bacterial and archaeal genomes: Improving accuracy and consistency. Chem Rev 2007;8:3431-344.

16. MAL-ED Network Investigators. Relationship between growth and illness, enteropathogens and dietary intakes in the first 2 years of life: findings from the MAL-ED birth cohort study BMJ Global Health 2017;2:e000370.

17. Faith JJ, McNulty NP, Rey FE, Gordon JI. Predicting a human gut microbiota's response to diet in gnotobiotic mice. Science 2011;333:101-04.

18. Dobin A, Davis CA, Schlsinger F, et al. STAR: ultrafast universal RNA-seq aligner. Bioinformatics 2013;1:15-2.

19. Liao Y, Smyth GK, Shi W. featureCounts: an efficient general purpose program for assigning sequence reads to genomic features. Bioinformatics 2014;30:923-30.

20. Wang L, Wang S, Li W. RSeQC: quality control of RNA-seq experiments. Bioinformatics 2012; 28:2184-5.

21. Kosek M, Haque R, Lima A, et al. Fecal markers of intestinal inflammation and permeability associated with the subsequent acquisition of linear growth deficits in infants. Am J Trop Med Hyg. 2013;88:390-396.

22. Kelly P, Bell L, Amadi B, et al. TAME trial: a multi-arm phase II randomised trial of four novel interventions for malnutrition enteropathy in Zambia and Zimbabwe – a study protocol. BMJ Open 2019;9:e027548.

23. Cardoso JF. Source separation using higher order moments. Proceedings of the ICASSP. 1989;2109-2112.

24. Saelans W, Cannoodt R, Sayes Y. A comprehensive evaluation of module detection methods for gene expression data. Nature Comm 2018; 9:1-12.

25. Plerou V, Gopikrishnan P, Rosenow B, Amaral LA, Guhr T, Stanley HE. Random matrix approach to cross correlations in financial data. Phys Rev E Stat Nonlin Soft Matter Phys 2002;65:1-18.

26. Strimmer K. fdrtool: a versatile R package for estimating local and tail area-based false discovery rates. Bioinformatics 2008;24:1461-62.

27. Alexa A., Rahnenfuhrer J. topGO: Enrichment Analysis for Gene Ontology. Bioconductor 2013.

28. Wei T, Simko V. R package “corrplot”: Visualization of a Correlation Matrix. 2017.

29. Anderson MJ. A new method for non-parametric multivariate analysis of variance. Austral Ecology 2001;26:32-46.

30. Oksanen J, Blanchet FG, Friendly M, et al. vegan: Community Ecology Package. 2019.

31. Tritos NA, Klibanski A. Effects of growth hormone on bone. Prog Mol Biol Trans Sci 2016; 138:193-212.

32. Hoeflich A, Pintar J, Forbes B. Current perspectives on insulin-like growth factor binding protein (IGFBP) research. Front Endocrinol 2018;9:667.

33. Quarles LD. Endocrine functions of bone in mineral metabolism regulation. J Clin Invest 2008;118:3820-28.

34. Wolff V, Aleil B, Giroud M et al. Soluble platelet glycoprotein V is a marker of thrombosis in patients with ischemic stroke. Stroke 2005;36:e17-e19

35. O'Sullivan S, Gilmer JF, Medina C. Matrix metalloproteinases in inflammatory bowel disease: an update. 2015;1-19

36. Gehrig JL, Venkatesh S, Chang HW, et al. Effects of microbiota-directed foods in gnotobiotic animals and undernourished children. Science 2019;365:aau4732.

37. Vivier E, Artis D, Colonna M, et al. Innate Lymphoid Cells: 10 Years On. Cell 2018;5:1054-6.

38. Kawabe T, Sun SL, Fujita T, et al. Homeostatic Proliferation of Naive CD4+ T Cells in Mesenteric Lymph Nodes Generates Gut-Tropic Th17 Cells. J Immunol 2013;190:5788-98.

39. Persson E, Uronen-Hansson H, Semmrich M, et al. IRF4 Transcription-Factor-Dependent CD103+CD11b+ Dendritic Cells Drive Mucosal T Helper 17 Cell Differentiation. Immunity 2013;38:958-69.

40. Schlitzer A, McGovern N, Teo P, et al. IRF4 Transcription Factor-Dependent CD11b+ Dendritic Cells in Human and Mouse Control Mucosal IL-17 Cytokine Responses. Immunity 2013;38:970-83.
